# Retrotransposon Expression Is Upregulated in Adulthood and Suppressed during Regeneration of the Limb in the Axolotl (*Ambystoma mexicanum*)

**DOI:** 10.1002/adbi.202400502

**Published:** 2025-05-29

**Authors:** Samuel Ruiz‐Pérez, Nicolás Alcaraz, Karla Torres‐Arciga, José Antonio Ocampo‐Cervantes, Alejandra Cervera, Clementina Castro‐Hernández, Cynthia Gabriela Sámano‐Salazar, Ernesto Soto‐Reyes, Rodrigo González‐Barrios

**Affiliations:** ^1^ Laboratorio de Regulación de la Cromatina y Genómica Instituto Nacional de Cancerología Av. San Fernando 22, Belisario Domínguez Sección XVI Tlalpan, Ciudad de México 14080 Mexico; ^2^ Protein Memory Program Novo Nordisk Foundation Center for Protein Research Faculty of Health and Medical Sciences University of Copenhagen Blegdamsvej 3B Copenhagen 2200 Denmark; ^3^ Danish Cancer Institute Strandboulevarden 49 Copenhagen 2100 Denmark; ^4^ Centro de Investigaciones Biológicas y Acuícolas de Cuemanco Universidad Autónoma Metropolitana, Unidad Xochimilco Antiguo Canal Cuemanco 3, Pista Olímpica Virgilio Uribe Xochimilco, Ciudad de México 16034 Mexico; ^5^ Laboratorio de Genómica Biomédica y Bioinformática Instituto Nacional de Medicina Genómica Periférico Sur 4809, Arenal Tepepan Tlalpan, Ciudad de México 14610 Mexico; ^6^ Departamento de Ciencias Naturales Universidad Autónoma Metropolitana, Unidad Cuajimalpa Vasco de Quiroga 4871, Contadero Cuajimalpa de Morelos, Ciudad de México 05348 Mexico; ^7^ Departamento de Biología Celular Facultad de Ciencias Ciudad Universitaria Coyoacán, Ciudad de México 04510 Mexico

**Keywords:** adulthood, axolotl, blastema, regeneration, retrotransposon

## Abstract

The axolotl (*Ambystoma mexicanum*) has a great capacity to regenerate its tissues; however, the fidelity and success of its regenerative process diminish with age. Retrotransposons make up the largest portion of the axolotl genome, and their expression may be involved in this age‐related decline. Through an integrative analysis of repetitive element expression using RNA sequencing, it is shown that *Ty3* retrotransposons are highly upregulated in the axolotl as an effect of chronological aging. Other non‐long‐terminal‐repeat transposons, including long interspersed nuclear element 1, function as hubs of gene coexpression networks involved in muscle development and regulation of apoptosis and connective tissue replacement, which are also suppressed in adulthood. By contrast, it is found that during regeneration of the limb, these pathways and the expression of *Ty3* retrotransposons are distinctly downregulated. Although the blastema can readjust most of the transposon differential expression in adulthood, several elements remain affected and may have an impact in the immune response during regeneration. This analysis provides a profile of retrotransposon expression through chronological aging and during limb regeneration in the axolotl and indicates that transposons are responsive to physiological changes in a tissue‐specific way and may participate in the gene coregulatory networks underlying the regenerative process.

## Graphical abstract

The axolotl's remarkable regenerative abilities decline with age, the causes may include the numerous repetitive elements within its genome. This study uncovers how *Ty3* retrotransposons and coexpression networks involving muscle and immune pathways respond to aging and regeneration, suggesting that transposons respond to physiological shifts and may play a role in gene coregulatory networks, offering fresh insights into regenerative biology.

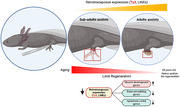

## 1 Introduction

The Mexican axolotl or *Ambystoma mexicanum* (Shaw and Nodder, 1789)^[^ ^1^ ^]^ is a salamander native to the canals and lakes of Xochimilco and Chalco, Mexico, that has been widely studied due to its great capacity to regenerate its cells, tissues, and whole‐body parts. Although *A. mexicanum* retains a remarkable regenerative capacity throughout its life, the rate, fidelity, and success of its regenerative process diminish with age.^[^ ^2^ ^]^ Among the many factors that could influence this decline are the increase in body and extremity size and mass, ossification of the skeleton, thickening and loss of flexibility of the skin, changes in nerve function, and shifts in circulating factors, hormones, immune response, or metabolism.^[^ ^2^ ^]^ However, the causes of this age‐related decrease in its regenerative capacity have not been fully clarified.

Until recently, research into the factors underlying regenerative biology has been mostly characterized by studies of protein‐coding genes.^[^ ^3^ ^]^ However, based on the most recent genome sequence assembly for the *d/d* strain (AmexG_v6.0‐DD), around 65% (≈18.6 Gb) of the axolotl genome consists of repetitive sequences.^[^ ^4^ ^]^ Moreover, the largest portion of the axolotl repeatome is composed of long terminal repeat (LTR) retroelements (≈26%, ≈7.2 Gb) of the *Ty3* type (≈11%, 3.2 Gb).

The numerous repetitive elements (REs) within the axolotl's ≈32 Gb genome are particularly interesting as molecular interactors with a potential role during regeneration. Of note, the activity of REs in several organisms is intricately linked to the innate immune system and the inflammatory response, whether triggered by infections, injuries, or other cellular stressors.^[^ ^5^ ^]^ Moreover, several studies have reported the negative effects of RE activity at both the genomic and physiological levels. Transposition and aberrant expression of inactive transposable elements (TEs) can introduce potentially catastrophic alternative gene‐splicing patterns and gene expression profile changes in some diseases,^[^ ^6^ ^]^ and thus silencing of TEs is a critical part of the maintenance of essential cellular homeostasis. By contrast, other studies have shown that the activity of some REs, especially TEs, can be beneficial to their hosts.^[^ ^7^ ^]^ For example, by regulating the expression of protein‐coding genes, retrotransposons can perform crucial functions in embryogenesis, such as affecting pluripotency and directing cell fate decisions.^[^ ^8^ ^]^ Nonetheless, the involvement of REs in animal regeneration has been addressed by only a few studies.^[^ ^3^, ^9^, ^10^, ^11^, ^12^, ^13^, ^14^ ^]^

For instance, Mashanov et al.^[^ ^10^ ^]^ have reported large‐scale changes in the transcriptional activity of LTRs during regeneration of radial organ complexes in the sea cucumber *Holothuria glaberrima*. Elewa et al.^[^ ^12^ ^]^ showed that both Gypsy and Harbinger transposons respond to injury in the adult salamander *Pleurodeles waltl*. In the axolotl, Zhu et al.^[^ ^11^ ^]^ reported both overexpression and higher retrotransposition of a long interspersed nuclear element 1 (*LINE‐1*) in the dedifferentiating tissues of the limb blastema. They showed that there was a dramatic upregulation of *LINE‐1* transcriptional activity as early as 2 days postamputation (dpa). They also reported that other putative TEs and *LINE‐1*‐related Piwi‐interacting RNAs (piRNAs) appear to be activated in the regenerative limb, with different dynamics. Although their results did not establish the role of these elements in tissue repair, they suggested that their reactivation is a ubiquitous phenomenon, and a marker for cellular dedifferentiation in the early stages of limb regeneration.

Studies in *A. mexicanum* and *H. glaberrima* have revealed differential retroelement expression during regeneration but differ in their interpretation of RE function after injury. Mashanov and Zueva^[^ ^3^ ^]^ proposed that changes in TE expression during regeneration may not simply reflect host transcriptional exploitation or reduced epigenetic silencing;^[^ ^11^ ^]^ instead, these changes could be precisely regulated at tissue, cell, and RE levels, suggesting REs may have unrecognized roles in postinjury tissue regrowth.

In this context, the physiological state of the organism could also be a crucial factor with an impact on the coordinated function of TEs during regeneration. Repetitive element transcriptional dysregulation might be a cause or effect of the shifts in circulating factors, immune response, or metabolism, which lead to the age‐related decrease in regenerative capacity in the axolotl.^[^ ^2^ ^]^ Indeed, studies in mice and other model organisms have shown that active TEs contribute to the aging process,^[^ ^15^, ^16^ ^]^ and both active and inactive elements also accumulate in age‐related neurodegenerative processes and diseases.^[^ ^17^ ^]^ Additionally, changes in global RE transcript levels have been reported to be a better marker of biological age than protein‐coding genes in both human fibroblasts and *Caenorhabditis elegans*.^[^ ^18^ ^]^ Also, RE derepression has been described in senescent cells^[^ ^19^ ^]^ and RE transcripts have been implicated in inflammation and oxidative stress,^[^ ^15^, ^20^ ^]^ two key contributors to aging and disease with positive and negative effects for regeneration. The underlying mechanisms by which RE transcripts affect these processes are not yet clear, but could involve activation of innate immune responses, which also play a central role in tissue repair.^[^ ^21^ ^]^ In summary, the role of REs amid the interplay of aging and reparative regeneration is still unclear. However, many studies so far have focused on known coding genes and overlooked the repetitive genetic material, which is coincidentally the majority of the genome of the axolotl.

In this study, we conducted RNA‐seq analyses to characterize the transcriptomic profiles of protein‐coding genes and REs in the regenerating limb of native (Xochimilco) Mexican axolotls from two different age groups. In addition, to discern if gene and RE expression is affected differently by adulthood in distinct tissue types, we integrated into the analysis 124 RNA‐seq samples from previously published studies. We report that REs, mainly of the *Ty3* superfamily, are predominantly upregulated in adulthood but downregulated during regeneration of the limb. Additionally, we identified several coexpression modules that suggest an indirect TE‐mediated regulatory network directing the immune, metabolic, and developmental responses during chronological aging and regeneration.

## 2 Results

### 2.1 A Major Suppression of Muscle Development and Function Underlies Limb Regeneration

**Figure 1:**
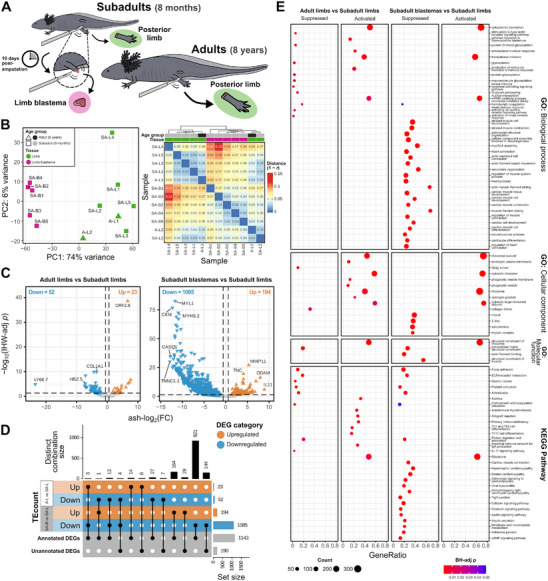
Gene expression differences between native axolotl samples of distinct age groups and tissues. A) Graphical representation of the tissue sampling of native axolotls, which consisted of five limb amputations and five limb blastemas (10 days postamputation) from subadult specimens (8 months old), and two limb amputations from adult specimens (8 years old). B) Left: principal component analysis (PCA) plot of gene and repetitive element counts (TEcount) after variance stabilizing transformation (VST). Right: heatmap of sample‐to‐sample distances (1 − Pearson correlation(VST[TEcount counts])). C) Volcano plots based on TEcount quantification show the gene expression differences between adult and subadult limbs and between subadult blastemas and subadult limbs. Genes with significantly upregulated (adaptive shrinkage (ash)‐log_2_(FC) > log_2_(1.5), independent hypothesis weighting (IHW)‐adj *p* < 0.05) or downregulated (ash‐log_2_(FC) < −log_2_(1.5), IHW‐adj *p* < 0.05) expression (differentially expressed genes (DEGs)) are represented by orange and blue triangles, respectively. D) UpSet plot of the total DEGs per contrast for TEcount quantification, their distinct combinations, and their annotation status. E) Dotplot shows the significantly activated or suppressed gene ontology (GO) terms and Kyoto Encyclopedia of Genes and Genomes (KEGG) pathways identified for these contrasts by fast gene set enrichment analysis (FGSEA). SA: subadult, A: adult, L: limb, B: limb blastema.

To determine the genes that are differentially expressed in adulthood and during limb blastema formation, we extracted RNA samples from the amputation of a posterior limb of five wild‐type male subadult axolotls (aged 8 months) and two wild‐type male adult axolotls (aged 8 years). Ten days postamputation, we collected blastema tissues only from the five subadult axolotls (**Figure** **1**A), since none of the adult axolotls displayed any development of regenerative tissue. This timepoint corresponds to the early blastema phase, which lasts until 38 dpa, according to previous reports.^[^ ^22^, ^23^, ^24^ ^]^ We subjected the samples to RNA‐seq, and quantified and analyzed their gene and RE expression levels following two distinct pipelines (see the Experimental Section and Figure S1 in the Supporting Information).

Principal component analysis (PCA) of the normalized counts revealed the presence of two subgroups within the dataset: limbs and limb blastemas (Figure 1B). Based on Pearson's correlation, a subadult limb tissue (SA‐L4) showed the highest sample‐to‐sample distance when contrasted with two subadult blastemas (SA‐B3 and SA‐B1). Differential expression analysis (DEA) revealed that 943–1085 of the differentially expressed genes (DEGs) in the contrast between subadult blastemas and subadult limbs were downregulated, and 137–194 genes were upregulated (Figure 1C,D and Figure S2C (Supporting Information)). Importantly, in total for all contrasts, 228 genes are not annotated in the *A. mexicanum* genome reference assembly v6.0‐DD (Figure 1D). Next, we conducted gene set enrichment analysis to identify which biological processes were enriched among all annotated genes. The subadult blastemas versus subadult limbs contrast showed a higher downregulation at the process level, with the suppression of 496–628 gene ontology (GO) terms and 50–51 Kyoto Encyclopedia of Genes and Genomes (KEGG) pathways and the activation of 9–308 GO terms and 0–18 KEGG pathways (Figure 1E and Figure S2D (Supporting Information)). The suppressed terms were mainly associated with muscle cell development and function, extracellular matrix signaling, and cellular junctions. By contrast, activated processes were related to ribosomal components and processes, translation, and mRNA catabolism.

### 2.2 Native Adult Axolotls Display an Activation of RNA and Ribosomal Processes

To identify the genes and biological processes generally impacted by adulthood (chronological aging of 88 months), we contrasted the limb samples from adult axolotls against the limb samples from subadult axolotls. Even though the total number of DEGs in this contrast was lower than the subadult blastemas versus subadult limb comparison, it similarly showed more underexpression, with 52–84 downregulated and 16–23 upregulated genes (Figure 1C,D and Figure S2C (Supporting Information)). Conversely, 16–20 GO terms and 0–19 KEGG pathways were activated, while 3–14 GO terms and 0–10 KEGG pathways were suppressed. The top suppressed terms were related to extracellular matrix constituents, coagulation, the Golgi lumen, and the stimulatory C‐type lectin receptor signaling pathway (Figure 1E and Figure S2D (Supporting Information)). As in the blastemas versus limbs contrast, activated terms in the age contrast included ribosomal element assembly, translation, mRNA catabolism, and protein targeting.

### 2.3 *Ty3* Expression Is Downregulated during Limb Regeneration

**Figure 2:**
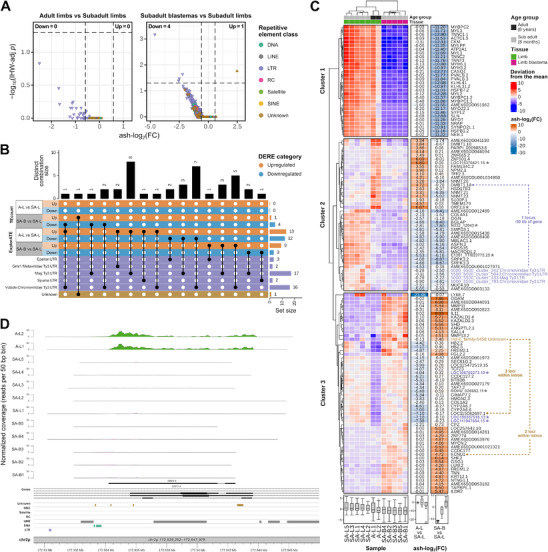
Repetitive element expression changes in the axolotl's adult and regenerating limbs. A) Volcano plots based on TEcount quantification show the repetitive element (RE) expression differences between adult and subadult limbs and between subadult blastemas and subadult limbs. REs with upregulated (ash‐log_2_(FC) > log_2_(1.5)) or downregulated (ash‐log_2_(FC) < −log_2_(1.5)) expression are represented by up‐pointing and down‐pointing triangles, respectively. Triangle color represents the RE class. B) UpSet plot of the total DEREs per contrast and quantification method (TEcount or ExplorATE), their distinct combinations, and their RE classification. C) Heatmap of the deviation from the mean of each VST‐normalized gene/RE count (TEcount), split into clusters by the global dendrogram. Columns with ash‐log_2_(FC) by contrast are shown on the right for each gene/RE. Genes with a genomic overlap or relative proximity to one or more loci of a DERE are connected with a dashed line. Elements which are annotated as genes but contain transposon‐related domains are marked in bold and with an asterisk (*). D) Karyoplot of *ORF2.6*, a transposon‐related locus which showed the highest upregulation in the limb as an effect of adulthood. RNA‐seq read coverage of each sample is plotted on top. SA: subadult, A: adult, L: limb, B: limb blastema.

Next, we quantified and analyzed RE expression at the subfamily level. The subadult blastema showed a few significantly differentially expressed RE subfamilies (DEREs), i.e., 3–4 downregulated and 1–9 upregulated, with an apparent trend of RE downregulation (**Figure** **2**A,B and S3C (Supporting Information)). Among these DEREs, 8 belonged to the *Chromoviridae* family and 6 to the *Mag* family, both members of the *Ty3* retrotransposon superfamily and the LTR class. One *Epsilon* and one *Spuma* subfamilies were also differentially expressed in the subadult blastema. In addition, one DERE has not been annotated in the reference RE library for *A. mexicanum* and pertains to an unknown class. For the contrast between adult limbs and subadult limbs, only one quantification method, ExplorATE, allowed the detection of DEREs, all of the LTR class (Figure 2B and Figure S3C (Supporting Information)).

To jointly compare the expression of genes and REs, we performed hierarchical clustering on the elements with the highest absolute log fold change for both the tissue and the age group contrasts. To this end, we calculated the amount by which each gene or RE deviated from its mean across all samples (Figure 2C). We found two main blocks of genes/REs that covaried across subadult blastemas and limbs; one included downregulated genes related to muscle development and function (cluster 1: *TNNC1.1*, *CASQ1*, *CKM*, *MYL2*, *KLHL41*, *SLN*, etc.) and the other connected an unknown upregulated RE (*rnd‐6_family‐8458*) with upregulated genes related to bone formation, remodeling, and regeneration (top and bottom cluster 3: interleukin 11 (*IL11*), *ODAM*, *SHF.2*, *KAZALD1.4*, *SHD*, *GSG1*, etc.).

For the age group contrast, we found a set of genes for which adult limbs had both lower expression (top cluster 3: *LY6E.7*, *LOC111947684.15*, *HBZ.5*, *LOC105357518.13*, *CYP2A6.6*, etc.) and higher expression (top cluster 2: *LOC115370421.16*, *ORF2.6*, *ZNF501.4*, *DMBT1.18*, *TMEM179*, etc.) than the subadult limbs. Conversely, we found a set of genes/REs that covaried across both tissues and age groups (*SRPK3*, *BGLAP, PRSS35*, *SMPD3*, and *ASPN.2*, etc.). These genes, together with four *Ty3* retrotransposons, are also downregulated in subadult blastemas when compared to subadult limbs.

In both the age and tissue contrasts we found several elements that, although containing predicted transposon protein domains, are included in the main genome annotation along with other coding and noncoding genes and thus not considered in the RE library. In other words, these are additional transposon‐related loci for which we found significant expression changes in the adult limb (LTRs: *LOC111947684.15*, *LOC105357518.13*, and non‐LTRs: *LOC115370421.16*, *ORF2.6*, *ROHU_026682.11*), and in the subadult blastema (non‐LTRs: *E1301_TTI023775.25*, *N332_12043*, etc.). In particular, *ORF2.6*, required for reverse transcription of *LINE‐1*, showed the highest upregulation in the limb as an effect of adulthood (Figures 1C and 2C,D).

Repetitive elements have shown considerable regulatory potential, specifically in the modification of gene expression. To address the possible role of the identified DEREs on gene regulation, we evaluated their genomic context and vicinity to DEGs. To do this, we annotated each locus of each DERE to its nearest genomic/genic feature. As shown in **Table** **1**, most DERE loci were located in intergenic regions and in the introns of non‐DEGs. We found that only one unknown DERE (*rnd‐6_family‐8458*) contained loci that both had a genomic overlap and shared a deviation‐from‐the‐mean cluster with DEGs (i.e., *KCNU1* and *LOC115082697.1*). Specifically, only two out of 5724 loci of *rnd‐6_family‐8458* were contained within an intron of *KCNU1*, a gene that was upregulated in the blastema. Two other *rnd‐6_family‐8458* loci were within an intron of *LOC115082697.1*, a gene that was downregulated in adult limbs (Figure 2C). These results suggest that the potential regulation of gene expression by REs in the subadult blastemas of native axolotls is mostly independent of their genomic proximity to the genes.

**Table 1:** Summary of the loci of differentially expressed repetitive element subfamilies and the genomic features with which they overlap for the subadult blastemas versus subadult limbs contrast of axolotl tissues. ▲: upregulated; ▼: downregulated; •: not differentially expressed in the subadult blastemas (SA‐B) versus subadult limbs (SA‐L) contrast. Note: differentially expressed elements in this table are the ones identified based on TEcount quantification.

Genomic/genic feature	DEG category (SA‐B vs SA‐L)	Shared heatmap cluster (Figure 2C)	Downregulated REs				Upregulated RE
			*5000_5500_cluster_433*	*5500_6000_cluster_5043*	*6000_6500_cluster_342*	*6000_6500_cluster_793*	*rnd‐6_family‐8458*
TSS‐promoter	▲	Not shared	.	.	.	.	.
	•	Not shared	5	.	.	.	7
	▼	Not shared	.	.	.	.	.
5′‐UTR	▲	Not shared	.	.	.	.	3
	•	Not shared	2	.	.	.	.
	▼	Not shared	.	.	.	.	1
Coding sequence (CDS)	▲	Not shared	.	.	.	.	.
	•	Not shared	.	.	.	.	4
	▼	Not shared	.	.	.	.	.
Intronic	▲	Shared	.	.	.	.	2
	▲	Not shared	12	2	4	2	60
	•	Shared	.	.	.	.	2
	•	Not shared	383	71	66	84	1565
	▼	Not shared	60	11	7	10	204
Exonic	▲	Not shared	.	.	.	.	.
	•	Not shared	3	.	.	.	18
	▼	Not shared	.	.	.	.	.
3′‐UTR	▲	Not shared	.	.	.	.	.
	•	Not shared	4	.	1	.	3
	▼	Not shared	.	.	.	.	1
TTS	▲	Not shared	.	.	.	.	.
	•	Not shared	.	.	.	.	11
	▼	Not shared	.	.	.	.	.
Other genic	▲	Not shared	.	.	.	.	.
	•	Not shared	1	1	.	.	.
	▼	Not shared	.	.	.	.	.
−5 kb	▲	Not shared	1	.	.	.	1
	•	Not shared	15	2	2	1	29
	▼	Not shared	2	.	.	.	2
+5 kb	▲	Not shared	.	1	.	.	1
	•	Not shared	10	2	2	1	44
	▼	Not shared	.	.	.	.	1
−10 kb	▲	Not shared	1	.	.	.	2
	•	Not shared	28	3	3	2	34
	▼	Not shared	.	.	.	.	3
+10 kb	▲	Not shared	1	.	.	.	2
	•	Not shared	14	2	2	4	42
	▼	Not shared	.	1	.	.	4
−50 kb	▲	Not shared	2	.	1	1	5
	•	Not shared	86	18	12	16	213
	▼	Not shared	5	5	.	.	9
+50 kb	▲	Not shared	2	.	1	.	4
	•	Shared	.	.	1	.	.
	•	Not shared	91	19	15	17	286
	▼	Not shared	6	1	1	1	23
Intergenic region			900	200	122	164	3138
Total loci per DERE			1634	339	240	303	5724

### 2.4 Age‐Related Muscle Development Downregulation in the Limb Replaces Muscle Suppression in the Blastema

**Figure 3:**
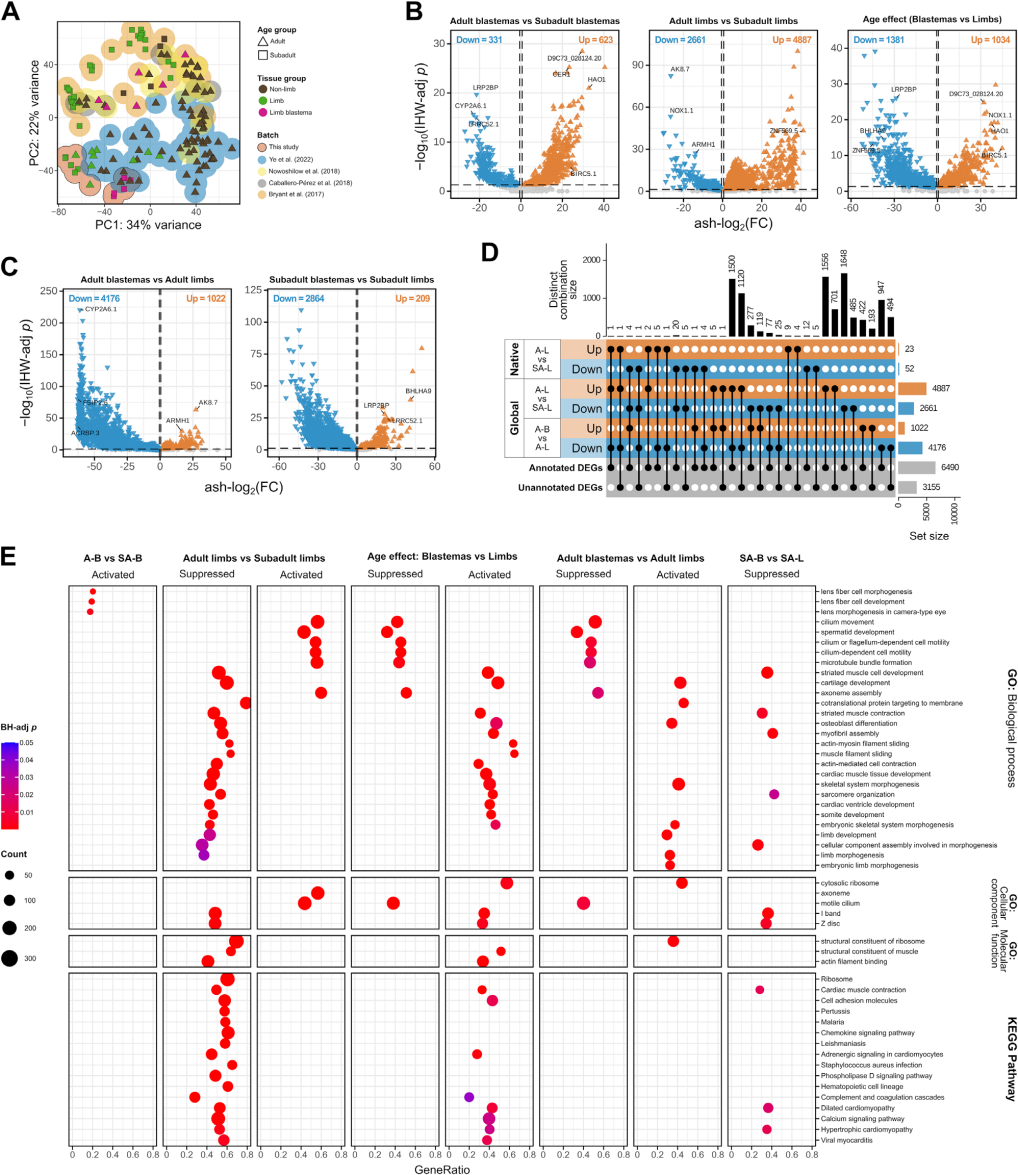
Gene expression differences between global axolotl samples of distinct age groups and between blastemas and limb tissues. A) PCA plot of gene and repetitive element counts (TEcount) after VST. B) Volcano plots based on TEcount quantification show the gene expression differences between adult and subadult blastemas, between adult and subadult limbs, and the age effect differences between these tissues. C) Volcano plots based on TEcount quantification show the gene expression differences between adult blastemas and adult limbs, and between subadult blastemas and subadult limbs. Genes with significantly upregulated (ash‐log_2_(FC) > log_2_(1.5), IHW‐adj *p* < 0.05) or downregulated (ash‐log_2_(FC) < −log_2_(1.5), IHW‐adj *p* < 0.05) expression (DEGs) are represented by orange and blue triangles, respectively. D) UpSet plot of the total DEGs per contrast, including the comparison of adult limbs versus subadult limbs for both native and global axolotl samples, the adult blastemas versus adult limbs contrast, their distinct combinations, and their annotation status. E) Dotplot shows the significantly activated or suppressed GO terms and KEGG pathways identified for each contrast by FGSEA. SA: subadult, A: adult, L: limb, B: limb blastema.

To determine if the expression of genes with roles in regeneration is affected differently by adulthood in distinct tissue types, we integrated into the analysis 124 previously published RNA‐seq samples that included nonlimb, limb, and limb blastema tissues, divided into the subadult and adult age groups^[^ ^4^, ^25^, ^26^, ^27^ ^]^ (Table S1 (Supporting Information)). Based on the PCA of all 136 samples (**Figure** **3**A), we conducted the subsequent DEAs considering batch effects.

We found that adult axolotls exhibit many DEGs compared to their subadult counterparts in all tissues evaluated (Figure 3B and Figure S4A (Supporting Information)). Moreover, based on TEcount quantification, 2622 (≈54%) of the 4887 genes that were upregulated in the contrast of adult limbs versus subadult limbs were also downregulated in the adult blastema, representing a ≈63% of the 4176 genes downregulated in adult blastemas when contrasted with adult limbs (Figure 3D). However, in terms of both quantity and type of enriched biological pathways, nonlimb and limb tissues from adult axolotls were much more similar to each other than to the adult blastema: adulthood in the limb and nonlimb tissues triggered a substantial suppression of pathways related to translation and muscle development and the activation of cell motility and cilium or flagellum formation (Figure 3E and Figure S4B (Supporting Information)).

Pairwise tissue contrasts revealed that during regeneration, the blastema exhibits a major downregulation of genes compared to the limb and nonlimb tissues (Figure 3C and Figure S4A (Supporting Information)). At the biological pathway level, the blastemas from adult axolotls show significant activation of the cytosolic ribosome, embryonic limb and skeletal system morphogenesis, and nonembryonic limb and cartilage development, when compared to the limb (Figure 3E). At the same time, they exhibit suppression of transposition pathways, cilium or flagellum‐dependent cell motility and spermatid development. The age‐related suppression of muscle, cartilage, and nervous structure development observed in the adult limb, appears to reach an almost equivalent effect, outside of the regenerative process, to the muscle suppression needed during the development of the blastema (Figure 3E).

### 2.5 Adult Axolotls’ Limbs Undergo a *Ty3* Retrotransposon Upregulation That Is Absent during Regeneration

**Figure 4:**
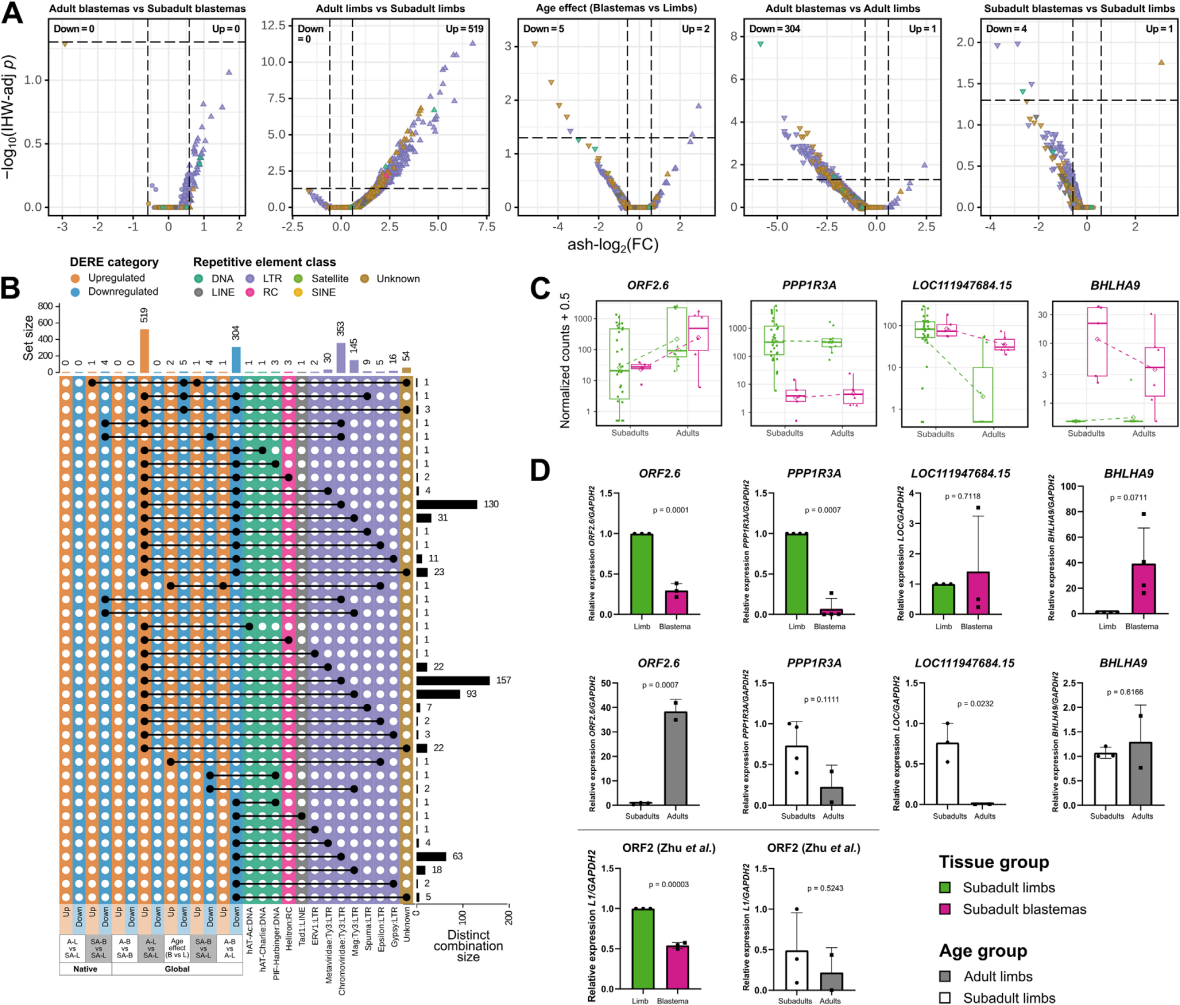
Effects of adulthood on the repetitive element expression changes in the axolotl's regenerating limb. A) Volcano plots based on TEcount quantification of global samples show the RE expression differences between age groups for blastemas and limbs, age effect differences between these tissues, as well as the contrasts between adult blastemas and adult limbs, and between subadult blastemas and subadult limbs. REs with upregulated (ash‐log_2_(FC) > log_2_(1.5)) or downregulated (ash‐log_2_(FC) < −log_2_(1.5)) expression are represented by up‐pointing and down‐pointing triangles, respectively. Triangle color represents the RE class. B) UpSet plot based on TEcount quantification shows the total DEREs per contrast, including only global axolotl samples, their distinct combinations, and their RE classification. C) Plots of the normalized TEcount counts of the genes and REs with the highest significant differential expression (|ash‐log_2_(FC)|) in the contrasts shown in Figure 3B,C and panel (A). Glyceraldehyde‐3‐phosphate dehydrogenase 1 *(GAPDH.1)* and *GAPDH.2* housekeeping genes are included as a reference for quantifying gene expression. Each filled square or triangle represents the count for a specific global sample. Dashed lines connect the means of different age groups of the same tissue group (empty rhombi). D) Quantitative polymerase chain reaction (qPCR) validation of selected REs (*ORF2.6*, *LOC111947684.15*, and *LINE‐1 ORF2* from Zhu et al.^[^ ^11^ ^]^) and protein‐coding genes (*PPP1R3A, BHLHA9*) at subadults versus adult limbs (*n* = 3 and *n* = 2, respectively) and subadult limbs and blastema limbs (*n* = 3) groups. Relative expression values were calculated under a 2^−ΔΔC^T model. Bars represent the average relative expression of each target gene in the target samples (adults or blastemas), normalized by *GAPDH.2* and calibrated by the reference samples (subadults or limbs, respectively). *t*‐tests were used to assess the significance of the difference between pairs of conditions. The error bars show the standard deviations and *p* values are shown at each graph.

Subsequently, we evaluated the expression of REs at the subfamily level in all samples. We found that adult axolotls exhibit a significantly higher expression of retrotransposons than their subadult counterparts in the limb and nonlimb tissues, but not in the blastema. When comparing adult and subadult limb tissues, we identified 0–24 downregulated and 223–519 upregulated DEREs, with most being *Ty3* retrotransposons or unknown REs (**Figure** **4**A and **Table** **2**). Similarly, the contrast of nonlimb tissues of different age groups showed 3–31 downregulated and 134–396 upregulated DEREs, of which the majority were *Ty3* or unknown REs (Figures S9A and S7B (Supporting Information) and Table 2). On the other hand, we did not detect any significant DEREs in the adult blastemas versus subadult blastemas comparison with TEcount (Figure 4A). Although we found 49 DEREs in this contrast with the ExplorATE quantification, this still represented several fewer DEREs than in the adult limb versus subadult limb contrast from the same method (Figure S7B, Supporting Information).

**Table 2:** Count and annotation of significantly differentially expressed repetitive element subfamilies across contrasts of age and tissue of axolotl samples. DERE: differentially expressed repetitive element subfamily; ▲: upregulated; ▼: downregulated. Note: counts of differentially expressed elements in this table correspond to the sum of both TEcount and ExplorATE quantifications.

Group of samples	Contrasted samples (estimated effects)	DERE category	DNA						LINE					LTR												RC		SINE		
			hAT‐Ac	hAT‐Blackjack	hAT‐Charlie	hAT‐Tip100	Other DNA	PIF‐Harbinger	L1	L2	Penelope	RTE‐X	Tad1	Bel‐Pao	Copia	DIRS	*Epsilon*	ERV1	ERVL	Foamy	Gmr1:*Metaviridae*:*Ty3*	*Mag*:*Ty3*	*Spuma*	*Ty3*	Vclade:*Chromoviridae*:*Ty3*	Helitron	Satellite	5S‐Deu‐L2	Unknown	TOTAL
Native	Adult limbs versus subadult limbs	▲	.	.	.	.	.	.	.	.	.	.	.	.	.	.	.	.	.	.	2	8	1	.	2	.	.	.	.	13
		▼	.	.	.	.	.	.	.	.	.	.	.	.	.	.	2	.	.	.	.	3	.	.	7	.	.	.	.	12
	Subadult blastemas versus subadult limbs	▲	.	.	.	.	.	.	.	.	.	.	.	.	.	.	1	.	.	.	.	3	.	.	5	.	.	.	1	10
		▼	.	.	.	.	.	.	.	.	.	.	.	.	.	.	.	.	.	.	.	3	1	.	3	.	.	.	.	7
Global	Adult blastemas versus adult limbs	▲	.	.	.	.	.	.	.	.	.	.	.	.	.	.	5	1	.	.	1	1	.	.	4	.	.	.	.	12
		▼	.	.	1	.	.	3	.	.	.	.	1	.	.	.	3	1	.	.	10	67	4	13	211	2	.	.	31	347
	Adult blastemas versus adult nonlimbs	▲	.	.	.	.	.	.	.	.	.	.	.	.	.	.	6	1	.	.	2	3	1	.	12	.	.	.	.	25
		▼	2	.	1	.	.	6	6	1	1	.	1	6	1	6	11	4	1	1	40	227	14	29	610	5	2	1	116	1092
	Adult limbs versus subadult limbs	▲	1	.	1	.	.	2	.	.	1	.	.	1	.	.	8	1	.	1	32	168	15	15	334	3	.	1	48	632
		▼	.	.	.	1	.	.	.	3	.	.	.	1	.	.	2	1	2	.	.	3	7	.	4	.	.	.	.	24
	Adult blastemas versus subadult blastemas	▲	.	.	.	.	.	.	.	.	.	.	.	.	.	.	4	.	.	.	1	10	2	.	12	.	.	.	.	29
																														
		▼	.	1	.	.	.	.	.	.	.	.	.	.	.	.	1	.	1	.	1	2	.	.	14	.	.	.	.	20
	Adult nonlimbs versus subadult nonlimbs	▲	.	.	1	.	.	2	1	.	.	.	.	1	.	.	5	1	.	1	17	135	6	7	246	3	.	1	44	471
		▼	.	.	.	.	.	.	.	1	.	1	.	.	.	.	5	1	3	.	1	3	5	.	11	.	.	.	.	31
	Age effect (blastemas versus limbs)	▲	.	.	.	.	.	.	.	.	.	.	.	.	.	.	6	.	.	.	1	1	.	.	4	.	.	.	.	12
		▼	.	.	.	.	.	.	.	.	.	.	.	.	.	.	1	.	.	.	4	14	1	.	14	.	.	.	4	38
	Age effect (blastemas versus nonlimbs)	▲	.	.	.	.	.	.	.	1	.	.	.	.	.	.	9	.	.	.	.	5	3	.	13	.	.	.	.	31
		▼	.	.	.	.	.	1	1	.	.	.	.	.	.	.	1	.	.	.	3	18	1	.	23	.	.	.	8	56
	Age effect (limbs versus nonlimbs)	▲	.	.	.	.	.	.	.	.	.	.	.	.	.	.	1	1	.	.	1	8	3	.	14	.	.	.	.	28
		▼	.	.	.	.	.	.	.	.	.	.	.	.	.	.	.	.	.	.	1	2	1	.	3	.	.	.	.	7
	Subadult blastemas versus subadult limbs	▲	.	.	.	.	.	.	.	.	.	.	.	.	.	.	1	.	1	.	1	7	3	.	10	.	.	.	1	24
		▼	.	.	.	.	.	1	.	.	.	.	.	1	.	.	3	.	.	.	.	8	3	.	2	.	.	.	.	18
	Subadult blastemas versus subadult nonlimbs	▲	.	1	.	.	.	.	.	.	.	.	.	.	.	.	.	.	1	.	1	6	.	.	10	.	.	.	1	20
		▼	.	.	.	.	.	.	.	.	.	.	.	.	.	.	3	1	.	.	.	9	3	.	15	.	.	.	.	31
	Subadult limbs versus subadult nonlimbs	▲	1	.	.	.	.	.	.	1	.	.	.	.	.	.	.	.	.	.	1	6	6	.	9	.	.	.	.	24
		▼	.	.	.	.	1	.	.	.	.	.	.	.	.	.	4	1	.	.	2	13	4	.	21	.	.	.	.	46

Tissue comparisons revealed that during regeneration the blastema undergoes a major downregulation of mostly *Ty3* retrotransposons of the *Chromoviridae* and *Mag* families, relative to samples of the limb and nonlimb tissues (Figure 4A and Figure S9A (Supporting Information)). Moreover, based on TEcount quantification, 209 (≈40%) of the 519 REs that were upregulated in the comparison between adult limbs and subadult limbs were also downregulated in the adult blastema, representing an ≈72% of the 304 total REs downregulated in adult blastemas when contrasted with adult limbs (Figure 4B).

To jointly analyze gene and RE expression, we performed hierarchical clusterings based on the deviation from the mean of each element, group by the contrasts of interest (Figures S8 and S9B, Supporting Information). In cluster 3 of the first dendrogram (Figure S8, Supporting Information), we found a set of covarying genes/REs affected by adulthood in the limb that makes it possible to associate several upregulated genes (e.g., *ZNF569.5*, *TESMIN*, *ARHGAP11A*, *BHLHA9*, *FERD3L*, etc.) and LTRs (e.g., *Chromoviridae*, *Mag*, *Metaviridae*, etc.), with various downregulated genes (e.g., *NOX1.1*, *MDY.2*, *BIRC5.1*, *AK8.7*, *MSGN1*, etc.). By contrast, for most of the genes/REs in this cluster, the increase in age has a significantly different effects in the blastema. For instance, for some genes aging has the opposite effect: genes such as *FERD3L*, *TESMIN*, and *H2BC1.1* are downregulated, while *MSGN1*, *BIRC5.1*, and *MDY.2* are upregulated. Other genes such as *REG1B.1*, *CRYGB.53*, *HAO1*, and a transposon DNA polymerase (*D9C73_028124.20*), are only affected by age in the blastema, and not in the limb. Additionally, we identified sets of genes/REs that covary in blastemas when compared to limb tissues. For example, one set consisted of genes with a distinct upregulation in the subadult blastema, e.g., *BHLHA9* and *AMEX60DD029513*, which showed no differential expression in the blastema of adult axolotls. In some cases, blastemas of aged axolotls might be unable to regulate the expression of regeneration‐associated genes. By contrast, DEREs in these clusters exhibit a less notable differential expression than the genes and form a distinct set of *Ty3* retrotransposons (Figure S8, Supporting Information). Altogether, these results suggest that the gene and RE regulation needed during regeneration of the limb can be affected in multiple directions by an increase in age, which can also be observed when comparing the normalized counts of the most differentially expressed genes/REs across age and tissue types (Figure 4C). Compared to the coding genes, most REs were downregulated in the blastemas of adult axolotls but upregulated by adulthood in the limb (Figure S8, Supporting Information), with some exceptions (Figure 4C).

Furthermore, across all global contrasts, the majority of DERE loci analyzed were located in intergenic regions (≈56.6%) or within the introns (≈12.2%) of non‐DEGs (Tables S2 and S3, Supporting Information). Only a limited fraction of all DERE loci were located in the introns of downregulated (≈11.8%) and upregulated (≈6.3%) DEGs, e.g., *7750_8250_cluster_73* (Figure 4C). However, only a small fraction of the DERE loci that colocalized with DEGs shared a deviation cluster with a DEG (≈0.006%). Overall, these results indicate that the potential regulation of gene expression by REs in both adulthood and regeneration is mostly independent of their genomic proximity to genes.

Based on the analyses of native and global axolotls, we selected among the most differentially expressed elements a *LINE‐1* (*ORF2.6*), a *Ty3* retrotransposon (*LOC111947684.15*), two protein‐coding genes (*PPP1R3A* and *BHLHA9*), and the *LINE‐1 ORF2* reported by Zhu et al.^[^ ^11^ ^]^ in tail regeneration for further validation with quantitative PCR on native axolotl samples (Figure 4D). We confirmed the observations from the RNA‐seq analysis, where the expression of *ORF2.6* and *PPP1R3A* is reduced in the blastema, while the *Ty3* (*LOC111947684.15*) and *BHLHA9* were overexpressed in the blastema. We also observed the same trends seen in the RNA‐seq data, where *ORF2.6* was upregulated in adult limbs, whereas *Ty3* (*LOC111947684.15*) was downregulated. *PPP1R3A* and *BHLHA9* expression differences between tissues were not statistically significant. Interestingly, we found that the *ORF2* (*LINE‐1*), which was reported by Zhu et al.^[^ ^11^ ^]^ to be upregulated in the tail blastema, exhibited the opposite pattern in the limb blastema, showing decreased expression, similar to *ORF2.6*. No significant changes were observed when comparing subadult and adult limbs for this element.

### 2.6 A Network of Retrotransposition and Muscle Development Genes Is Jointly Suppressed in Adult Axolotls

**Figure 5:**
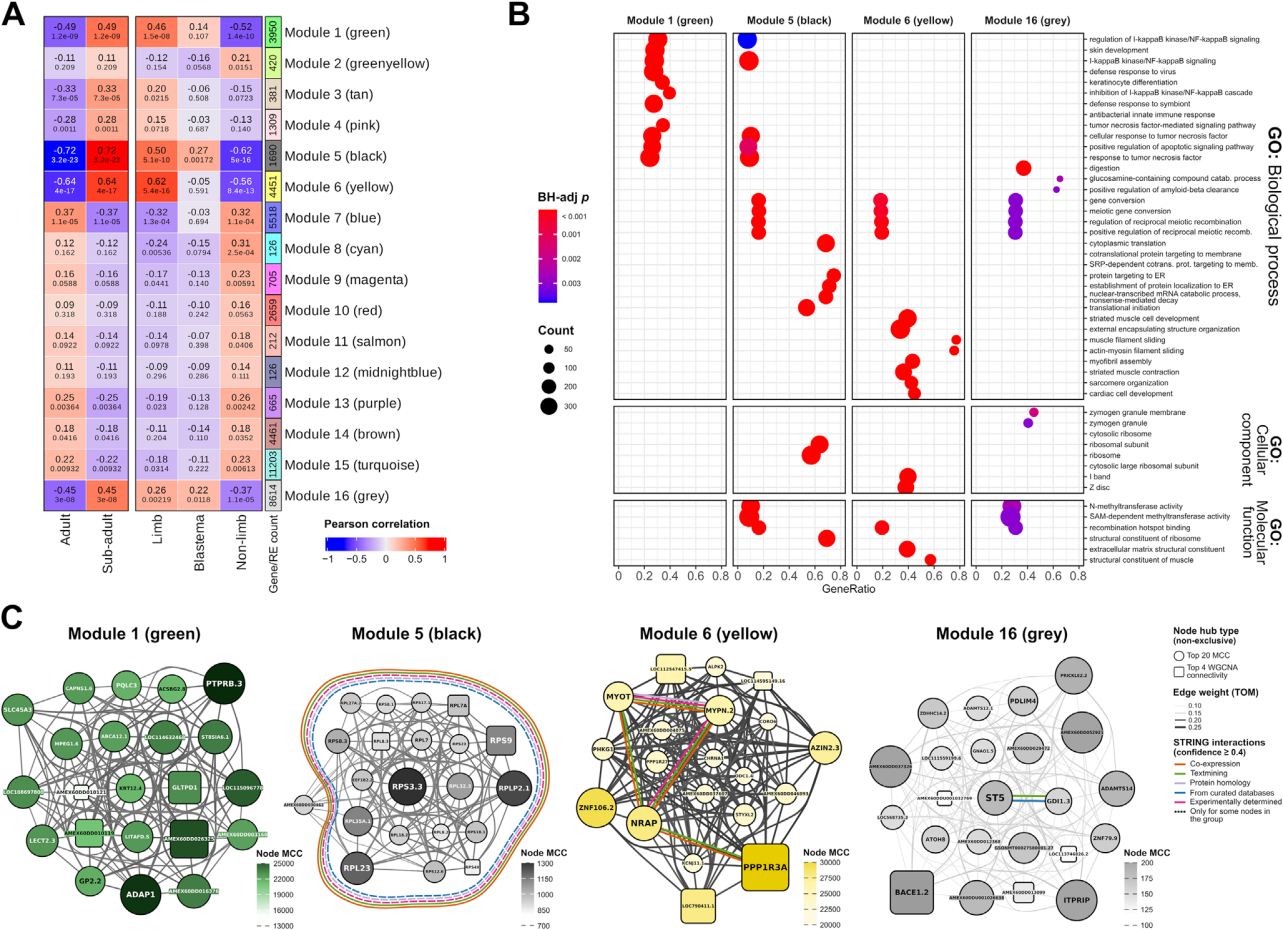
Gene and repetitive element coexpression and their association with adulthood and regeneration in the axolotl. A) Heatmap of module–trait Pearson correlation. Each cell shows the correlation value on top, and the associated BH‐adj *p* below it. B) Dotplot shows the significantly enriched GO terms identified by overrepresentation analysis (ORA) for each coexpression module. C) Coexpression subnetworks show the hub genes/REs in modules 1 (green), 5 (black), 6 (yellow), and 16 (gray), which were selected based on intramodular connectivity and maximal clique centrality (MCC) scores. Reported functional and physical protein associations are shown as color‐coded edges.

Besides their direct impact on the expression of colocalized or neighboring genes, DEREs may also establish expression networks with other protein‐coding genes, contributing to specific biological functions.^[^ ^28^ ^]^ To further explore the potential roles of REs in adulthood and in the development of the regenerative tissue of the limb, we examined the correlation between genes and REs of all samples using weighted gene coexpression network analysis (WGCNA). This analysis revealed 16 coexpression modules, with sizes ranging from 126 to 11 203 genes/REs (**Figure** **5**A).

To understand the physiologic significance of the modules, we correlated the 16 modules’ eigengenes with the sample traits: age group (adults or subadults) or tissue (nonlimb, limb, or blastema) (Figure 5A). The eigengenes of several modules were highly correlated with the adult age group trait, including module 5 (*r* = −0.72, BH‐adj *p* = 1.3 × 10^−21^), module 6 (*r* = −0.64, BH‐adj *p* = 7.9 × 10^−16^), module 1 (*r* = −0.49, BH‐adj *p* = 8.5 × 10^−9^), and module 16 (*r* = −0.45, BH‐adj *p* = 1.7 × 10^−7^). We found that these modules were also highly correlated with the tissue trait, specifically the limb tissues, although only two of these modules were slightly correlated with the limb blastema trait: module 5 (*r* = 0.27, BH‐adj *p* = 0.00552), and module 16 (*r* = 0.22, BH‐adj *p* = 0.027). Only two of these modules contain coexpressed REs: module 1 incorporated the *13750_14250_cluster_243*:*Spuma*:LTR and *5000_5500_cluster_187*:*Mag*:*Ty3*:LTR subfamilies, while module 16 included the *6250_6750_cluster_443*:*Chromoviridae*:*Ty3*:LTR subfamily and an unknown RE (*rnd‐3_family‐511*) (Figure S10A, Supporting Information). Table S4 (Supporting Information) presents the list of the DEGs and DEREs with the highest rate of change based on the differential expression contrasts, as well as their WGCNA module.

Then, to quantify the associations of individual genes/REs with our traits of interest (age group or blastema tissue), we measured their gene significance (GS) and module membership (MM). We found that GS and MM were highly correlated in modules 5 (black), 6 (yellow), and 1 (green), illustrating that genes/REs significantly associated with the age group of the axolotl were also the most important elements of their modules (Figure S10B, Supporting Information). However, modules associated to the blastema tissue showed a minimal GS–MM correlation (Figure S10C, Supporting Information).

Next, we conducted overrepresentation analysis (ORA) to determine whether these modules were significantly enriched for known gene ontologies (Figure 5B). Among the 681 significantly enriched pathways that we detected in module 5 (black), we found that the top ones were related to ribosome components, RNA metabolism, and translation. Module 6 (yellow) showed an overrepresentation of 1416 GO terms, mainly associated with muscle morphogenesis and function, extracellular matrix constituents, and chondrocyte differentiation. We detected a set of 1820 terms significantly enriched in module 1 (green), in which the predominant pathways were implicated in immune and inflammatory responses, negative regulation of NFκB, positive regulation of apoptotic signaling, cell survival and proliferation, and skin/keratinocyte development. Module 16 (gray) was significantly enriched for 60 GO terms, including digestion, *N*‐methyltransferase activity, and upregulation of connective tissue replacement.

Subsequently, to pin down the hub elements in each module, we selected the genes/REs with the highest intramodular connectivity (Figure 5C and Figure S10B,C (Supporting Information)) and maximal clique centrality (MCC) scores (Figure 5C). Most hub genes in module 5 (black) encoded for proteins of the small and large ribosomal subunits (RPSs and RPLs), as well as a eukaryotic translation elongation factor (*EEF1B2*), and the unannotated gene *AMEX60DD030462*. In addition, we annotated the functional and physical interactions of the hub genes based on the STRING database and observed that all proteins in the module except for the unannotated gene have reported interactions. In module 6 (yellow), the gene with the highest MCC score was *PPP1R3A*, a regulatory glycogen‐targeting subunit of protein phosphatase‐1 (PP1) which is essential for cell division, regulation of glycogen metabolism, and muscle function.^[^ ^29^ ^]^ Other hub genes in this module were also related to muscle development and activity (*NRAP*, *ZNF106*, *MYOT*, etc.). Moreover, we found three hub genes that encode for protein domains characteristic of retrotransposable elements. Specifically, *LOC790411* contains *LINE‐1* endonuclease reverse transcriptase and transposase domains, *LOC114595149* contains an exonuclease–endonuclease–phosphatase and a non‐LTR retrotransposon/retrovirus reverse transcriptase (RT), and *LOC112547415* contains a *DIRS‐1* RT domain and a *DIRS‐1* ribonuclease HI. These findings indicate that several retrotransposons might serve as hubs and indirectly participate in the gene co‐expression network of muscle development regulation and function that is suppressed by adulthood in the axolotl. Based on MCC, the top hub gene in module 1 (green) was *PTPRB*, which plays an important role in blood vessel remodeling during embryonic development, maintenance, and angiogenesis.^[^ ^30^ ^]^ Beta‐secretase 1 (*BACE1*), a proteolytic processor of the amyloid precursor protein (APP),^[^ ^31^ ^]^ was the top hub gene in module 16 (gray), which showed a significant positive correlation with the regenerative tissue. We also found a high interaction with *PRICKLE2*, a neural development gene involved in the inhibition of WNT/PCP/JNK signaling activated by APP.^[^ ^32^ ^]^ Interestingly, we also detected hub genes with LTR (*LOC113746826*) and non‐LTR (*LOC568735*) protein domains, and a potential regulator of their RNA pol II‐specific transcription (*ZNF79*). These findings suggest that retrotransposons and transcription factors might serve as hubs in the networks of connective tissue replacement and amyloid proteolysis that are activated during regeneration in the axolotl.

## 3 Discussion

Multiple studies have identified protein‐coding genes with a role in regeneration of the limb in *A. mexicanum*. Here, we have shown that there is a trend of gene downregulation between 10 dpa blastemas and limbs of native subadult axolotls, mainly represented by muscle development suppression, which corroborates previous studies.^[^ ^33^, ^34^, ^35^ ^]^ We found that ribosomal pathways were activated in the regenerative tissue. The management of ribosomes as key for maintaining differentiated cell identity and achieving successful cell regeneration after injury has been reported by previous studies.^[^ ^36^, ^37^, ^38^ ^]^ We additionally identified a significant upregulation of genes which have been previously implicated in limb regeneration in the axolotl. For instance, *IL11* exhibited significant upregulation in the regenerative tissue, possibly due to its role in the inflammatory response^[^ ^39^ ^]^ or in the limitation of fibrotic scarring during tissue regeneration.^[^ ^40^ ^]^ However, our results show that *IL11* has a role at 10 dpa, meaning it is differentially expressed beyond 3–5 dpa, as described previously.^[^ ^33^ ^]^ We also found that basic helix–loop–helix family member A9 (*BHLHA9*), involved in the regulation of apoptosis and apical ectodermal ridge formation during autopod development,^[^ ^41^, ^42^ ^]^ is also significantly upregulated in the limb blastema. To our knowledge, *BHLHA9* does not have a reported role in the axolotl blastema and is therefore a promising candidate for future investigations into the apoptotic process during limb regeneration in *A. mexicanum*.

Although there is a growing recognition of the role of TEs in development, their contribution to animal regeneration has just started to receive attention.^[^ ^3^ ^]^ In the case of the axolotl, Zhu et al.^[^ ^11^ ^]^ have described both overexpression and higher retrotransposition of *LINE‐1* in dedifferentiating tissues of the blastema. While they suggest that the reactivation of *LINE‐1* may serve as a marker for cellular dedifferentiation in the early stages of limb regeneration, they do not propose a precise function. Here, we characterized the expression profiles of all currently modeled REs in the regenerating limb of *A. mexicanum*. We showed that in the blastemas (10 dpa) of native (Xochimilco) axolotls, only one *Epsilon*, three *Mag*, and five *Chromoviridae* LTR retrotransposons, and one unannotated repeat exhibited signs of significant upregulation. Additionally, we report that only three *Mag*, 3 *Chromoviridae*, and one *Spuma* LTRs are downregulated in the native blastema. Among the most differentially expressed genes in the blastema, we found two loci with putative non‐LTR domains. However, in contrast with the results of Zhu et al.,^[^ ^11^ ^]^ we did not detect significant upregulation of any subfamily of *LINE‐1* in the blastema of native axolotls, but we did in the context of adulthood. Moreover, through quantitative polymerase chain reaction (qPCR) validation, we found that the *LINE‐1 ORF2* which was reported by Zhu et al.^[^ ^11^ ^]^ to be upregulated in the tail blastema, exhibited the opposite pattern in the limb blastema, and no significant changes were observed when comparing subadult and adult limbs for this element.

By incorporating axolotl tissue samples from multiple studies, we showed that during regeneration the blastema undergoes a major downregulation of mostly *Ty3* retrotransposons relative to the limb samples. These findings agree with the idea that differential expression of retroelements upon the development of the regenerate is a large‐scale phenomenon.^[^ ^3^ ^]^ Nonetheless, our observation of a predominant downregulation of REs is not concordant with the traditional view of RE dysregulation during regeneration, in which transposons profit from the global injury‐induced chromatin activation and transcriptional derepression (that leads to a germline‐like state) to amplify themselves in the host cell.^[^ ^3^, ^11^ ^]^ Instead, the suppression of *Ty3* transposons we describe here suggests a much more finely tuned epigenetic regulation of transposon expression during limb regeneration.

Several studies in multiple models have reported that REs accumulate progressively with age. For instance, studies in mice, flies, and other organisms have shown that active or transposable RE can contribute to the aging process,^[^ ^15^, ^16^ ^]^ and RE transcript accumulation is involved in age‐related neurodegenerative diseases.^[^ ^17^ ^]^ Until now, there has been no research into how chronological aging affects RE expression in *A. mexicanum*. The analyses we present here demonstrate a pattern comparable to that reported for other species,^[^ ^18^ ^]^ in which the limbs and other nonlimb tissues of the axolotl undergo a significant age‐related RE upregulation, mostly dominated by LTR retrotransposons of the *Ty3* superfamily. This profile of *Ty3* upregulation can reflect a higher transcription of TE mRNAs by the host's RNA polymerase II or an increase in the number of genomic insertions of the TE cDNA. Schneider et al.^[^ ^16^ ^]^ have revealed that in *Drosophila* endogenous overexpression of TEs during aging does not necessarily lead to new genomic insertions. Moreover, the presence of TE mRNAs or even DNA intermediates, and not insertion, may be the detrimental factor to cell and animal function. This could also be the case for the age‐related TE upregulation observed in the axolotl, however, these two scenarios are indistinguishable in our results.

Retrotransposon activity can affect gene‐regulatory networks and introduce both beneficial and catastrophic events to the host genome through several mechanisms.^[^ ^43^ ^]^ TE‐derived sequences can also undergo co‐option as effector proteins. These processes can lead to the gene expression changes that underlie some diseases.^[^ ^6^ ^]^ Our comparison of native adult axolotls against their subadult counterparts revealed an age‐related upregulated gene/TE cluster involving a *LINE‐1* transcript (*ORF2.6*), a non‐LTR retrotransposon (*LOC115370421.16*), *DMBT1.18*, *NNMT*, and two DNA‐binding TFs (*ZNF665.2* and *ZNF501.4*). *ORF2* is particularly interesting because it provides the RT activity required for target‐primed reverse transcription of the *LINE‐1* mRNA.^[^ ^44^ ^]^ The *KRAB*‐zinc finger protein *ZNF665* is also noteworthy, since previous work has revealed that many *KRAB*‐zinc finger proteins (KZFPs) recognize TEs or TE‐embedded sequences as genomic targets and can suppress them or facilitate the co‐option of their regulatory potential for the benefit of the host.^[^ ^45^ ^]^ In fact, in humans, *ZNF665* has targets of the *LINE‐1* family.^[^ ^45^ ^]^ Overall, the joint upregulation of these genes could mean that an age‐induced upregulation of *NNMT* might cause a reduction of the methylation levels in KZFPs binding sites within *LINE‐1*/TE promoters, thus reducing the affinity for KZFPs and their repressive potential over TEs. This would potentially generate an upregulation of KZFPs to counteract TE upregulation.

Besides the *Ty3* LTR predominant upregulation caused or affected by age, we have also shown that a number of *Ty3* and other non‐LTR retrotransposons are downregulated by adulthood. For instance, some loci with predicted non‐LTR retrotransposon domains (*LOC790411.1*: *LINE‐1* RTs and transposases, and *LOC112547415.5*: *DIRS‐1* RTs and ribonucleases) act as secondary hubs of a muscle development gene expression network that is jointly downregulated during adulthood in the axolotl. The main interactor in this network is *PPP1R3A*, the regulatory subunit of a glycogen‐associated form of PP1 that mediates muscle contraction and synthesis. Interestingly, we also found several downregulated non‐*KRAB ZNFs* (e.g., *ZNF106.2*) and KZFPs (e.g., *ZNF133*, *ZNF160*, *ZNF182*) in the network, suggesting an integrated regulation of non‐LTR retrotransposons and PP1‐related functions by TFs. Evolutionarily recent KZFPs almost universally target TEs, often have paralogs, and display a protein interactome that represses transcription by forming a heterochromatin‐inducing complex based on *TRIM28*,^[^ ^45^, ^46^ ^]^ another PP1 interactor involved in muscle function. The analysis of this expression network, hints at an interaction between three PP1‐related proteins (*PPP1R3A*, *PPP1R27*, *TRIM28*/*PPP1R157*), *ZNF* TFs, and non‐LTR retrotransposon expression. Hence, we suggest that a major factor in the suppression of skeletal muscle development and function observed during adulthood in the axolotl can be the downregulation of *PPP1R3A* expression and, with it, the limitation of its glycogen binding activity to PP1. Considering that in humans 95% of the promoter sequence of *PPP1R3A* is derived from LTR‐TE sequences,^[^ ^47^ ^]^ and since KZFPs are able to recognize TE‐embedded sequences and facilitate their regulatory potential,^[^ ^45^ ^]^ the downregulation of several *ZNF* TFs could have a direct detrimental effect on the expression and activity of *PPP1R3A*. Still, further studies in the axolotl are needed to establish the origin and RE composition of the *PPP1R3A* promoter sequence and its potential as a TF binding site for *C2H2‐ZNFs*. Similarly, *ZNF* TF repression may hinder the expression of the other muscle development genes or retrotransposons (*LINE‐1* and *DIRS‐1*) that we found downregulated in the module.

In general, in both chronological aging and regeneration, we found a significant differential expression of numerous *C2H2‐ZNFs* and KZFPs coupled with differentially expressed LTR and non‐LTR retrotransposons, also forming coexpression networks. We propose that KZFPs and other *C2H2‐ZNFs* may be important regulators of retrotransposon expression during adulthood and regeneration in the axolotl. Of note, the potential of TFs, specifically *KRAB* ZFPs, to play a major role in TE activity regulation during tissue regeneration has been hypothesized by Angileri et al.^[^ ^5^ ^]^

We have shown that more retrotransposons and protein‐coding genes are downregulated in the blastema of aged axolotls than in the blastema of younger axolotls. Moreover, ≈40% of the retrotransposons and ≈54% of the protein‐coding genes that were upregulated by chronological aging in the limb, were then downregulated in the blastema. This means that, while there is a significant counter response to previous age‐related gene and RE differential expression during the development of the regenerate, there are still several genes and REs that remain affected and may be impacting the regenerative process. In summary, in this study we provide a collection of genes and REs in the axolotl that are significantly differentially expressed as an effect of chronological aging or throughout the development of the blastema of the limb. We reveal that transposons, mostly of the *Ty3* superfamily, are predominantly upregulated as an effect of adulthood in the limb of *A. mexicanum*. By contrast, during regeneration of the limb, *Ty3* retrotransposon expression is largely downregulated. Overall, we argue that transposon expression changes during reparative regeneration in the axolotl do not represent a simple exploitation of the transcriptional machinery of the host, but rather a finely tuned response to the physiological state before and after an injury, specifically controlled at the tissue level.

### 3.1 Limitations of the Study

Even though we have shown that REs exhibit a clear trend of upregulation as an effect of chronological aging in the axolotl, further transcriptomic studies with specimens of more precise age groups (e.g., one group per year of age) could provide the means to define if RE transcript levels are a good marker of biological age in *A. mexicanum*,^[^ ^18^ ^]^ as well as to reveal the intermediate dynamics and physiological effects of RE expression through adulthood. Also, in the context of regeneration, analysis of RE expression in blastemas of diverse organs at more hourly or daily intervals after injury may facilitate the determination of the mechanisms by which retrotransposons regulate gene expression, metabolism, or immune response at the various stages of tissue formation. However, due to the critically endangered state of the native *A. mexicanum* and the strict regulations on experimentation on captive native axolotls, especially the ones of older age, sample acquisition for these kinds of studies is limited.

Our findings indicate that RE expression dynamics and regulation in the axolotl can also be specific to the particular insertion or locus of the repetitive element. Thus, RE transcript quantification in this organism should ideally be approached at the locus level. However, current RE quantification tools such as TEtranscripts provide more reliable and reproducible results when estimating combined abundances for all insertions of an element, i.e., at the subfamily level.^[^ ^48^ ^]^ In addition, our attempts at generating an index for the >33 million insertions of the axolotl repeatome suitable for TElocal using the nonparallelized script TElocal_indexer^[^ ^49^ ^]^ in a server with ≈1 TB of RAM resulted in a running time of more than 123 days before termination. This reflects the need for novel locus‐level expression quantification tools for repeatomes of tens of gigabases.

Furthermore, in the present study, we integrated RNA‐seq samples from native (Xochimilco) axolotls and specimens of various laboratory strains. This allows for generalization at the species level but overlooks the importance of the genotype of the axolotl on its physiology or regenerative process. For instance, there has been phenotypic evidence indicating that neither aging nor repetitive amputations have an important effect on the regenerative potential of urodeles, while other studies argue they can be detrimental.^[^ ^50^ ^]^ We have documented here and in a previous study^[^ ^51^ ^]^ that two 8 years old Xochimilco axolotls did not regenerate the hindlimb even after their first amputation. This opposing evidence may be due to the strain under study: impairment of regeneration after repeated amputations has been reported for American strains, and not for European strains.^[^ ^50^ ^]^ Moreover, the hybridization and prolonged breeding history of the main laboratory strain used today may have selected against developmental or metabolic processes involved in regeneration, as reported for spontaneous metamorphosis.^[^ ^52^, ^53^ ^]^ One additional limitation is that the available genome assembly for the axolotl was generated from a hybrid of *A. mexicanum* and *Ambystoma trigrinum*, which may or may not introduce differences in genomic analyses compared to observations made in the native axolotl growing under endemic conditions. Overall, these reports highlight the importance of taking the strain and genotype into account for aging and regeneration analyses in the axolotl and stress the need for advancing genomic and transcriptomic studies in native Xochimilco axolotls.

## 4 Experimental Section

### Sample Collection

Sample extraction was conducted as described previously.^[^ ^51^ ^]^ All biological samples were obtained from a captive population of Xochimilco Mexican axolotls (*A. mexicanum*) residing at the Unidad de Manejo Ambiental (UMA), Centro de Investigaciones Biológicas y Acuícolas de Cuemanco (CIBAC), Universidad Autónoma Metropolitana – Unidad Xochimilco (UAM‐X), Mexico City, Mexico. This captive population of *A. mexicanum* at the UMA, CIBAC was established in 2007 from 31 founder organisms of wild origin (from their habitat in Xochimilco) and careful reproduction was conducted to prevent endogamy or inbreeding.^[^ ^54^ ^]^

Sedation of the axolotls was accomplished through a 20 min immersion in a tank containing benzocaine at a concentration of 50 mg L^−1^ prior to the amputation process. Biological samples were meticulously collected, using a stereoscopic microscope, from the metatarsi of the (posterior) limbs of five male wild‐type subadult axolotls (aged 8 months), as well as from two male wild‐type adult axolotls (aged 8 years). Ten days after the amputation, blastema tissues were collected only from the five subadult axolotls, since none of the adult axolotls displayed development of blastema tissue even after 6 months of monitoring. In total, 12 tissue samples were collected. No animals were sacrificed for the purpose of this study, and all the axolotls were safely reintroduced to their habitat at the UMA, CIBAC following the final sample collection.

### RNA Extraction and Sequencing

The collected tissues were preserved in RNAlater (Invitrogen), at 4 °C for a maximum of 24 h prior to processing. The RNA was extracted by using TRIzol Reagent (Invitrogen, CA, USA). Ribosomal RNA depletion was performed using the RiboZero Gold kit (Illumina, CA, USA). Library construction was performed based on the manufacturer's recommendation for SmarterStranded V2 kit (Takara Bio, CA, USA). The final library quantity was measured by KAPA SYBR FAST qPCR and library quality was evaluated by TapeStation D1000 ScreenTape (Agilent Technologies, CA, USA). Paired‐end sequencing was carried out on an Illumina HiSeq sequencer (Illumina, CA, USA) with a read length configuration of 150 bp and 30 million reads per sample. Of these, quality assessment with FastQC 0.12.1^[^ ^55^ ^]^ and MultiQC 1.19^[^ ^56^ ^]^ showed zero sequences flagged as having poor quality, and thus all raw reads were included in posterior analyses.

### External RNA‐Seq Datasets

In addition to the 12 RNA‐seq datasets from native axolotl samples collected in this study, 124 datasets from previously published studies were also analyzed.^[^ ^4^, ^25^, ^26^, ^27^ ^]^ Each dataset was assigned a tissue group (nonlimb, limb, or limb blastema) and an age group (adult or subadult) based on its available metadata. For samples for which there was no described age group between these two, the age group was assigned based on the age of sexual maturation reported for the axolotl.^[^ ^2^ ^]^ Male axolotls 9 months old or younger and females 12 months old or younger were labeled as subadults; older axolotls were determined to be adults. The metadata of these datasets are presented in Table S1 (Supporting Information).

### Genomic Element Annotation

The axolotl reference genome (AmexG_v6.0‐DD), transcriptome (AmexT_v47‐AmexG_v6.0‐DD), proteome (AmexT_v47_protein), and their annotations were downloaded from https://www.axolotl‐omics.org/assemblies.^[^ ^4^ ^]^

Protein‐coding sequences from the proteome file were functionally annotated using eggNOG‐mapper 2.1.12^[^ ^57^ ^]^ based on eggNOG 5.0 orthology data.^[^ ^51^ ^]^ Sequence searches were performed using DIAMOND 2.1.8^[^ ^58^ ^]^ in ultrasensitive mode. Queries were realigned with HMMER 3.3.2^[^ ^59^ ^]^ to the PFAM^[^ ^60^ ^]^ domains found on the orthologous groups.

Transcripts in the transcriptome annotation (GTF) file were functionally reannotated for GO terms, KEGG pathways, and NCBI/Entrez Gene IDs by querying their transcript_name(s) against the genome‐wide annotations of human (org.Hs.eg.db),^[^ ^61^ ^]^ mouse (org.Mm.eg.db),^[^ ^62^ ^]^ and *Xenopus* (org.Xl.eg.db).^[^ ^63^ ^]^ All queries for each transcript were merged, duplicates were removed, and each transcript was assigned a comprehensive set of terms. Transcript annotation was grouped by gene_id, duplicates were removed, and each gene was assigned a set of GO ids and KEGG pathway ids. Each gene was assigned a gene_name (symbol) by selecting among the list of transcript_name(s) for the gene, with the following priority: 1) first [hs] transcript_name, 2) first transcript_name annotated by eggNOG‐mapper, 3) first [nr] transcript_name, 4) first [no identifier] transcript_name.

The reference RE library (AmexG_v6.0‐DD_Repeats) was retrieved from the UCSC Genome Browser track data hub at https://www.axolotl‐omics.org/trackhubs/AmexG_v6.0‐DD/Repeats/hub.description.^[^ ^4^ ^]^

### RNA‐Seq Data Analysis

Gene and RE expression were quantified following two independent approaches. In the first approach, all the RNA‐seq datasets were aligned to the axolotl reference genome using STAR 2.7.11a^[^ ^64^ ^]^ with parameters – outFilterMultimapNmax 200 – winAnchorMultimapNmax 400 – outFilterMismatchNoverLmax 0.04 – outFilterType BySJout. Gene and RE family expression was then quantified with TEcount under TEtranscripts 2.2.3^[^ ^48^ ^]^ with parameters – stranded no – mode multi and the reference gene (AmexT_v47‐AmexG_v6.0‐DD) and RE (AmexG_v6.0‐DD_Repeats) annotations. Quantification results for all samples were joined and imported as a count matrix. In the second approach, quantification was performed on the RNA‐seq datasets separately using Salmon 1.10.2^[^ ^65^ ^]^ for transcript expression and ExplorATE v0.1b^[^ ^66^ ^]^ for RE expression. Using tximport 1.26.0,^[^ ^67^ ^]^ transcript and RE locus abundances were imported and summarized to the gene and RE subfamily levels, respectively.

### Gene and Repetitive Element Differential Expression Analysis

The imported quantification data were analyzed for differential expression using DESeq2 1.38.0^[^ ^68^ ^]^ with two different designs. In the first design, the 12 native axolotl samples were analyzed independently under two additive models – Age_group and Tissue_group. In the second design, both native and previously published samples were analyzed under a single interaction model considering batch effect – Batch + Age_group + Tissue_group + Age_group:Tissue_group. Two‐tailed threshold‐based Wald tests of significance were performed using altHypothesis = “greaterAbs,” lfcThreshold = log2(1.5). Independent filtering and *p*‐value adjustment were performed using independent hypothesis weighting (IHW 1.26.0^[^ ^69^ ^]^ with a significance cutoff *α* = 0.05. Logarithmic fold changes were shrunk using adaptive shrinkage (ash)^[^ ^70^ ^]^ with a lfcThreshold = log2(1.5). DEGs or DEREs of interest for a specific contrast were defined as those presenting |ash‐log_2_(FC)| > log_2_(1.5) and IHW‐adj *p* < 0.05. Since two quantification approaches were used for both gene and RE expression, results were presented as ranges (e.g., “2–6 downregulated genes…”). Volcano plots were generated with EnhancedVolcano 1.16.0.^[^ ^71^ ^]^ Heatmaps and Upset plots were generated with ComplexHeatmap 2.14.0.^[^ ^72^ ^]^ Karyoplots were generated with karyoploteR 1.24.0.^[^ ^73^ ^]^

### Repetitive Element Genomic Context Annotation

All DERE loci coordinates were extracted from the repeat annotation file (AmexG_v6.0‐DD_Repeats) and annotated to their nearest genomic/genic features as recorded in the transcriptome (AmexT_v47‐AmexG_v6.0‐DD) using UROPA 4.0.3^[^ ^74^ ^]^ with the following priority: 1) inferred TSS‐promoter (−1000 bp, +100 bp from gene's 5′); 2) inferred TTS (‐100 bp, +1000 bp from gene's 3′); 3) coding sequence (CDS); 4) 5′‐UTR; 5) 3′‐UTR; 6) other exonic; 7) intronic; 8) other genic; 9) 5 kb upstream of gene; 10) 5 kb downstream of gene; 11) 10 kb upstream of gene; 12) 10 kb downstream of gene; 13) 50 kb upstream of gene; 14) 50 kb downstream of gene; 15) intergenic region.

### Gene Set Enrichment Analysis Based on Differential Expression

The complete gene ash‐log_2_(FC) lists that resulted from each contrast of the DEA were subjected to fast gene set enrichment analysis (FGSEA)^[^ ^75^ ^]^ under the GSEA function of clusterProfiler 4.6.0,^[^ ^76^ ^]^ using parameters exponent = 1, minGSSize = 10, maxGSSize = 500, eps = 0, pvalueCutoff = 0.05, pAdjustMethod = “BH,” nPermSimple = 1 000 000.

### Gene and Repetitive Element Coexpression Analysis

Gene and RE counts from the TEcount quantification of all samples were subjected to batch effect adjustment with ComBat‐seq of sva 3.46.0,^[^ ^77^ ^]^ specifying age group and tissue as biological covariates to preserve. The adjusted counts were subjected to variance‐stabilizing transformation (VST)^[^ ^68^, ^78^ ^]^ and used as input for coexpression analysis, which was performed using the WGCNA) R package v1.71.^[^ ^79^ ^]^ The *blockwiseModules* function of WGCNA was used to construct the network and group the genes and REs into coexpression modules in one step. The parameters entered were power = 8, networkType = “signed,” deepSplit = 2, pamRespectsDendro = F, minModuleSize = 30, reassignThreshold = 0, mergeCutHeight = 0.25, maxBlockSize = 52 000. The module eigengenes were then correlated with the phenotypes (age groups or tissues) using the *cor* (Pearson) function of WGCNA. The vectors of *p* values were generated using the corPvalueStudent function of the same package, and then fitted with the Benjamini–Hochberg method. GS and MM scores and correlations were defined and calculated as per the WGCNA R package.^[^ ^79^ ^]^

### Overrepresentation Enrichment Analysis for Coexpression Modules

The enrichment of biological pathways in each coexpression module was examined with overrepresentation analysis based on the hypergeometric test, as implemented in the *fgseaORA/fora* function of FGSEA 1.24.0.^[^ ^75^ ^]^ The GO annotation generated previously was used to produce a list of GO ids and their corresponding axolotl genes to employ as reference. Each module's enrichment was analyzed independently: the set of query genes was defined as all the genes detected by WGCNA for the module, and the gene universe/background was defined as the set of genes for which at least 3 samples of any of the traits significantly correlated to the module (BH‐adj *p* < 0.05) had a raw TEcount count of at least 10. The minimal and maximal sizes of a gene set to test were set to 10 and 500 genes, respectively.

### Hub Gene and Repetitive Element Detection

The hub genes/REs of the coexpression modules were identified using two ranking methods. First, a modified version of the *chooseTopHubInEachModule* function (WGCNA package) was used to pinpoint the four genes with the highest connectivity in each module. Second, the topological overlap matrix of each module was entered into the *exportNetworkToCytoscape* function (WGCNA package) to export each weighted network to node and edge files, with an adjacency threshold of 0.02. The nodes and edges were imported to Cytoscape 3.10.1^[^ ^80^ ^]^ and the top 20 hub genes/REs within each module were identified based on their MCC score as calculated with cytoHubba 0.1.^[^ ^81^ ^]^ The union set of both ranking methods was used to generate a new network on which the following algorithms were applied for visualization: edge‐weighted spring embedded layout (based on weight/TOM), yFiles (v1.1.3) Remove Overlaps and Organic Edge Router.^[^ ^82^ ^]^ Next, the Cytoscape StringApp 2.0.2^[^ ^83^ ^]^ was used to retrieve the protein associations (with a confidence score ≥ 0.4) between the identified hub genes from the STRING database.^[^ ^84^ ^]^

### Quantitative PCR

Total RNA was isolated using TRIzol reagent (Thermo Fisher Scientific), samples were collected from three 8 months old and two 8 years old axolotls by technical triplicate as specified by the manufacturer and cDNA was synthesized from 100 ng of RNA using the High Capacity cDNA Reverse Transcription Kit (Applied Biosystems/Thermo Fisher, 4374966) with random primers. Quantitative PCR was performed using Maxima SYBR Green/ROX qPCR Master Mix (Thermo Scientific, K0221) in a QuantStudio 3 (Applied Biosystems). The expression was normalized using the glyceraldehyde‐3‐phosphate dehydrogenase 2 (*GAPDH.2*) mRNA. The metadata of the primers used are listed in Table S5 (Supporting Information). The expression by the double delta CT (2^−ΔΔC^T) model was evaluated with a *n* ≥ 3 and *t*‐tests were used to assess the significance of the difference between pairs of conditions, 2^−ΔΔC^T.

### Statistical Analysis

Preprocessing of data, sample size, statistical methods used to assess significant differences between groups and the software used were described for each sample group in the previous Experimental Section subsections.

## Conflict of Interest

The authors declare no conflict of interest.

## Author Contributions

Conceptualization: S.R.‐P., N.A., R.G.‐B. Data curation: S.R.‐P. Formal analysis: S.R.‐P. Funding acquisition: C.G.S.‐S., E.S.‐R., and R.G.‐B. Investigation: S.R.‐P., K.T.‐A. Methodology: S.R.‐P. Project administration: C.G.S.‐S., E.S.‐R., and R.G.‐B. Resources: J.A.O.‐C., A.C., C.C.‐H., E.S.‐R., and R.G.‐B. Software: S.R.‐P. Supervision: N.A., E.S.‐R., and R.G.‐B. Validation: S.R.‐P., K.T.‐A. Visualization: S.R.‐P. Writing – original draft preparation: S.R.‐P., N.A., and R.G.‐B. Writing – review and editing: S.R.‐P., N.A., K.T.‐A., A.C., E.S.‐R., R.G.‐B.

## Supporting information



Supporting Information

Supplemental TableS1

Supplemental TableS2

Supplemental TableS3

Supplemental TableS4

Supplemental TableS5

## Data Availability

The raw RNA‐seq data from the native axolotl samples was deposited in the Gene Expression Omnibus under accession GSE237864. The external RNA‐seq datasets are available under accessions GSE92429, GSE182746, PRJNA354434 and PRJNA378982. All the code for the bioinformatic analyses of the RNA‐seq data is detailed in the following GitHub repository: https://github.com/samuelruizperez/axoloTE. The datasets resulting from these analyses are available in the following Zenodo repository: https://doi.org/10.5281/zenodo.10728952.
