# Supplementary material for: Retrotransposon Expression Is Upregulated in Adulthood and Suppressed during Regeneration of the Limb in the Axolotl (Ambystoma mexicanum)
Source: Adv Biol (Weinh). 2025 May 29;9(10):e00502. doi: 10.1002/adbi.202400502 (PMC12517315; doi:10.1002/adbi.202400502)
Supplement: Supplementary file 1 — Supporting Information [file ADBI-9-e00502-s002.pdf]

Supporting information

Retrotransposon expression is upregulated in adulthood and suppressed during regeneration of the limb in the axolotl (Ambystoma mexicanum)

Samuel Ruiz-Pérez[1], Nicolás Alcaraz[5], Karla Torres-Arciga[1], José Antonio Ocampo-Cervantes[2], Alejandra Cervera[3], Clementina Castro-Hernández[1], Cynthia Gabriela Sámano-Salazar[4], Ernesto Soto-Reyes[4]* and Rodrigo González-Barrios[1,6]*

[1] Laboratorio de Regulación de la Cromatina y Genómica, Instituto Nacional de Cancerología, Av. San Fernando 22, Belisario Domínguez Sección XVI, Tlalpan, 14080 Mexico City, Mexico.

[2] Centro de Investigaciones Biológicas y Acuícolas de Cuemanco, Universidad Autónoma Metropolitana, Unidad Xochimilco, Antiguo Canal Cuemanco 3, Pista Olímpica Virgilio Uribe, Xochimilco, 16034 Mexico City, Mexico.

[3] Subdirección de Genómica Poblacional, Instituto Nacional de Medicina Genómica, Periférico Sur 4809, Arenal Tepepan, Tlalpan, 14610 Mexico City, México.

[4] Departamento de Ciencias Naturales, Universidad Autónoma Metropolitana, Unidad Cuajimalpa, Vasco de Quiroga 4871, Contadero, Cuajimalpa de Morelos, 05348 Mexico City, Mexico.

[5] Protein Memory Program, Novo Nordisk Foundation Center for Protein Research, Faculty of Health and Medical Sciences, University of Copenhagen, Blegdamsvej 3B, 2200 Copenhagen, Denmark.

[6] Departamento de Biología Celular, Facultad de Ciencias, Universidad Nacional Autónoma de México, Investigación Científica, Ciudad Universitaria, Coyoacán, 04510 Mexico City, Mexico.

* **Corresponding authors**

E-mail: rodrigop@ciencias.unam.mx (RG-B)
E-mail: esotoreyes@cua.uam.mx (ES-R)

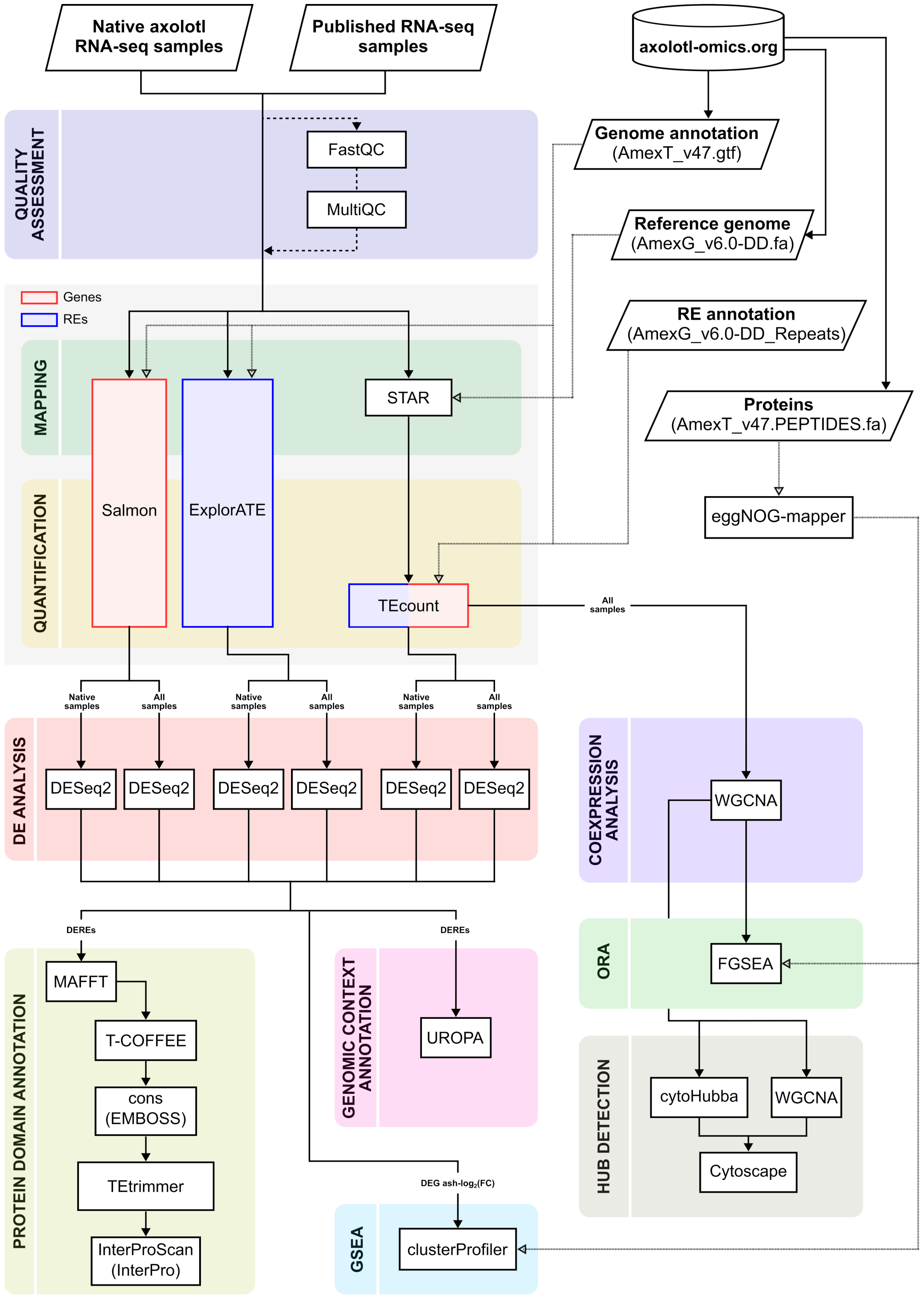

Figure S1. Flowchart of the bioinformatic pipelines used to analyze the RNA-seq samples of this study. Previously published axolotl RNA-seq samples and samples of native axolotls (this study) were analyzed following two distinct quantification approaches for both genes and repetitive elements. Differential expression, enrichment, and coexpression steps are included, as well as the annotation of certain genomic elements. RE: repetitive element; DE: differential expression; DERE: differentially expressed RE; DEG: differentially expressed gene; GSEA: gene set enrichment analysis; ORA: overrepresentation analysis; ash-log$_2$(FC): ashr-shrunken log fold change.

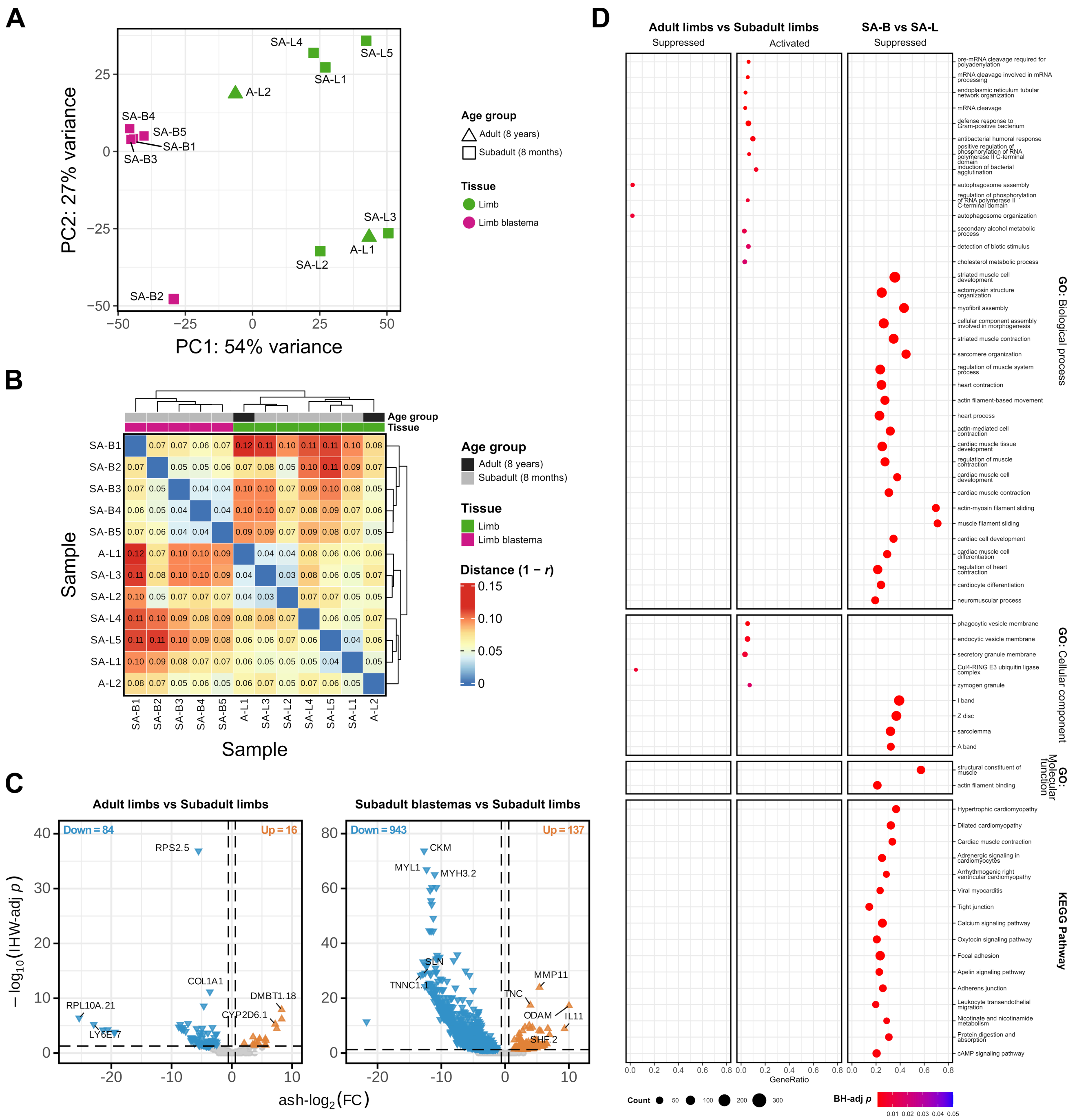

Figure S2. Gene expression differences between native axolotl samples of distinct age groups and tissues (Salmon quantification). (A) Principal component analysis (PCA) plot of gene counts (Salmon) after variance stabilizing transformation (VST). **(B)** Heatmap of sample-to-sample distances (1 − Pearson correlation(VST[Salmon counts])). **(C)** Volcano plots based on Salmon quantification show the gene expression differences between adult and subadult limbs and between subadult blastemas and subadult limbs. Genes with significantly upregulated (ash-log$_2$(FC) > log$_2$(1.5), IHW-adj p < 0.05) or downregulated (ash-log$_2$(FC) < −log$_2$(1.5), IHW-adj p < 0.05) expression (DEGs) are represented by orange and blue triangles, respectively. **(D)** Dotplot shows the significantly activated or suppressed gene ontology (GO) terms and Kyoto Encyclopedia of Genes and Genomes (KEGG) pathways identified for these contrasts by fast gene set enrichment analysis (FGSEA). SA: subadult, A: adult, L: limb, B: limb blastema.

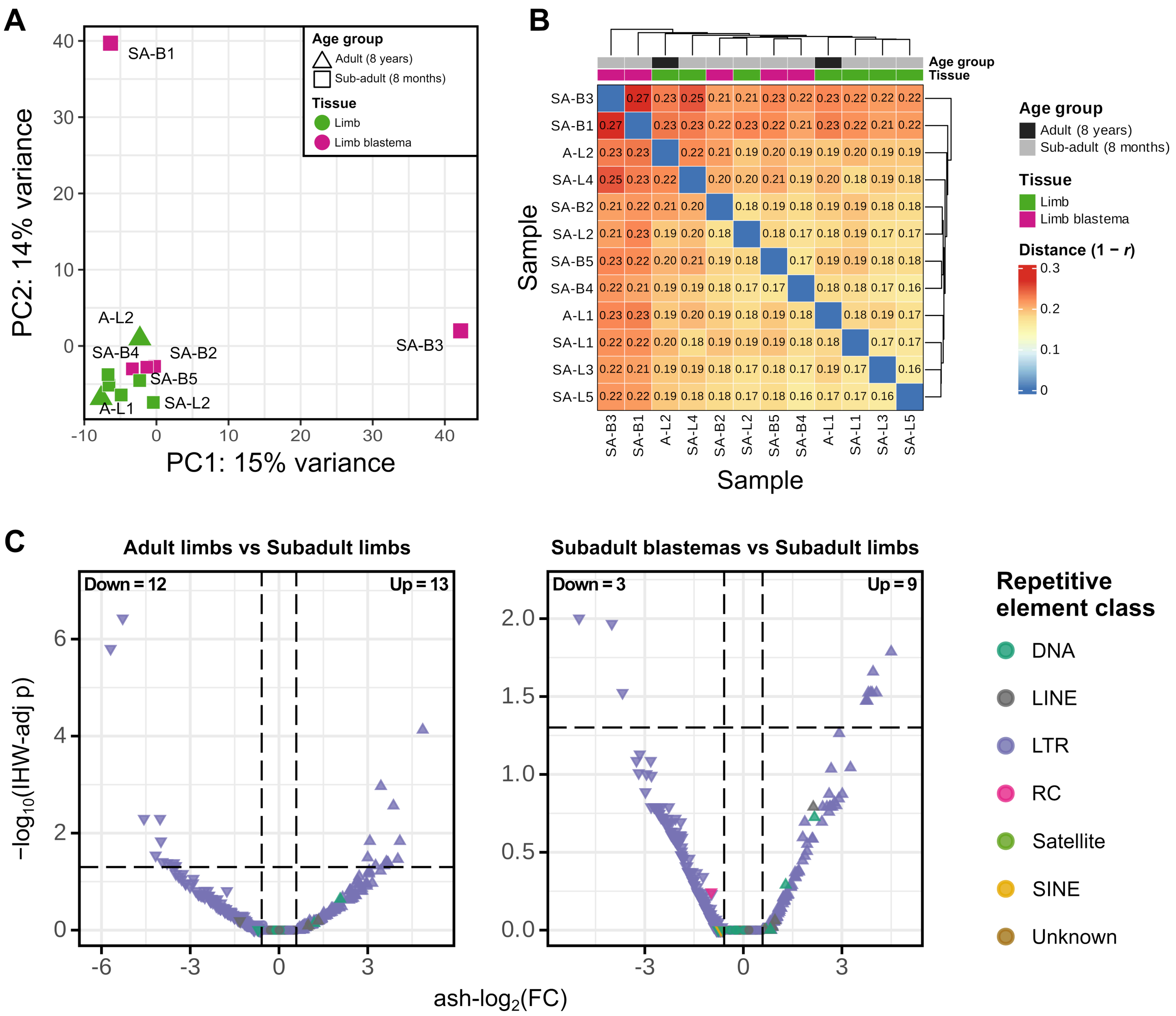

Figure S3. Repetitive element expression changes in the axolotl's adult and regenerating limbs (ExplorATE quantification). (A) Principal component analysis (PCA) plot of repetitive element subfamily counts (ExplorATE) after variance stabilizing transformation (VST). **(B)** Heatmap of sample-to-sample distances (1 − Pearson correlation(VST[ExplorATE counts])). **(C)** Volcano plots based on ExplorATE quantification show the repetitive element (RE) expression differences between adult and subadult limbs and between subadult blastemas and subadult limbs. REs with upregulated (ash-$\log_2$(FC) > $\log_2$(1.5)) or downregulated (ash-$\log_2$(FC) < −$\log_2$(1.5)) expression are represented by up-pointing and down-pointing triangles, respectively. Triangle color represents the RE class. SA: subadult, A: adult, L: limb, B: limb blastema.

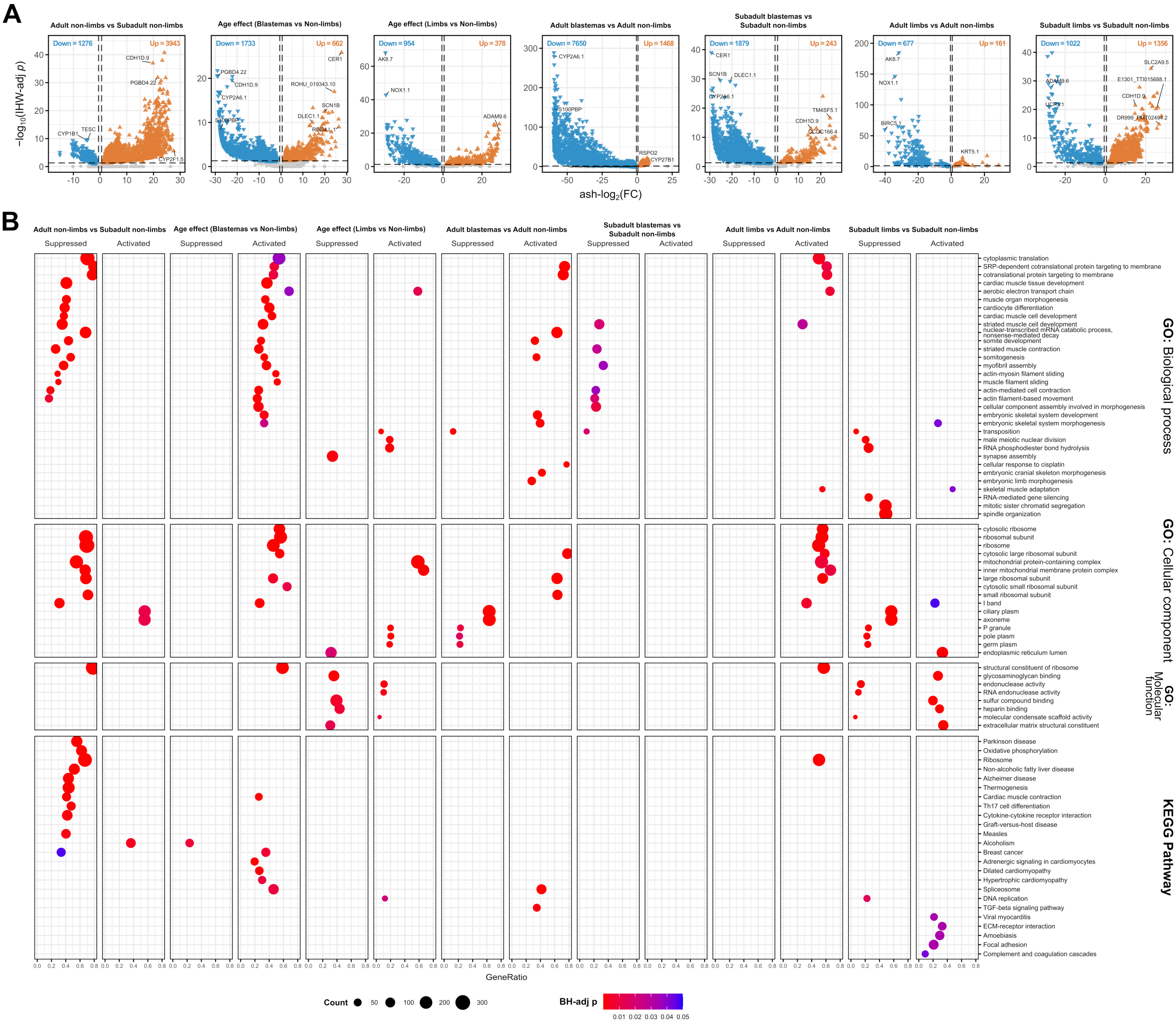

Figure S4. Gene expression differences between global axolotl samples of non-limb tissues and limbs and blastemas of distinct age groups. (A) Volcano plots based on TEcount quantification show the gene expression differences between adult and subadult non-limbs and the age effect differences between non-limbs and blastemas or limbs. Other tissue and age group contrasts involving non-limb tissues are also included. Genes with significantly upregulated (ash-$\log_2$(FC) > $\log_2$(1.5), IHW-adj p < 0.05) or downregulated (ash-$\log_2$(FC) < $-\log_2$(1.5), IHW-adj p < 0.05) expression (DEGs) are represented by orange and blue triangles, respectively. **(B)** Dotplot shows the significantly activated or suppressed gene ontology (GO) terms and Kyoto Encyclopedia of Genes and Genomes (KEGG) pathways identified for these contrasts by fast gene set enrichment analysis (FGSEA). SA: subadult, A: adult, L: limb, B: limb blastema.

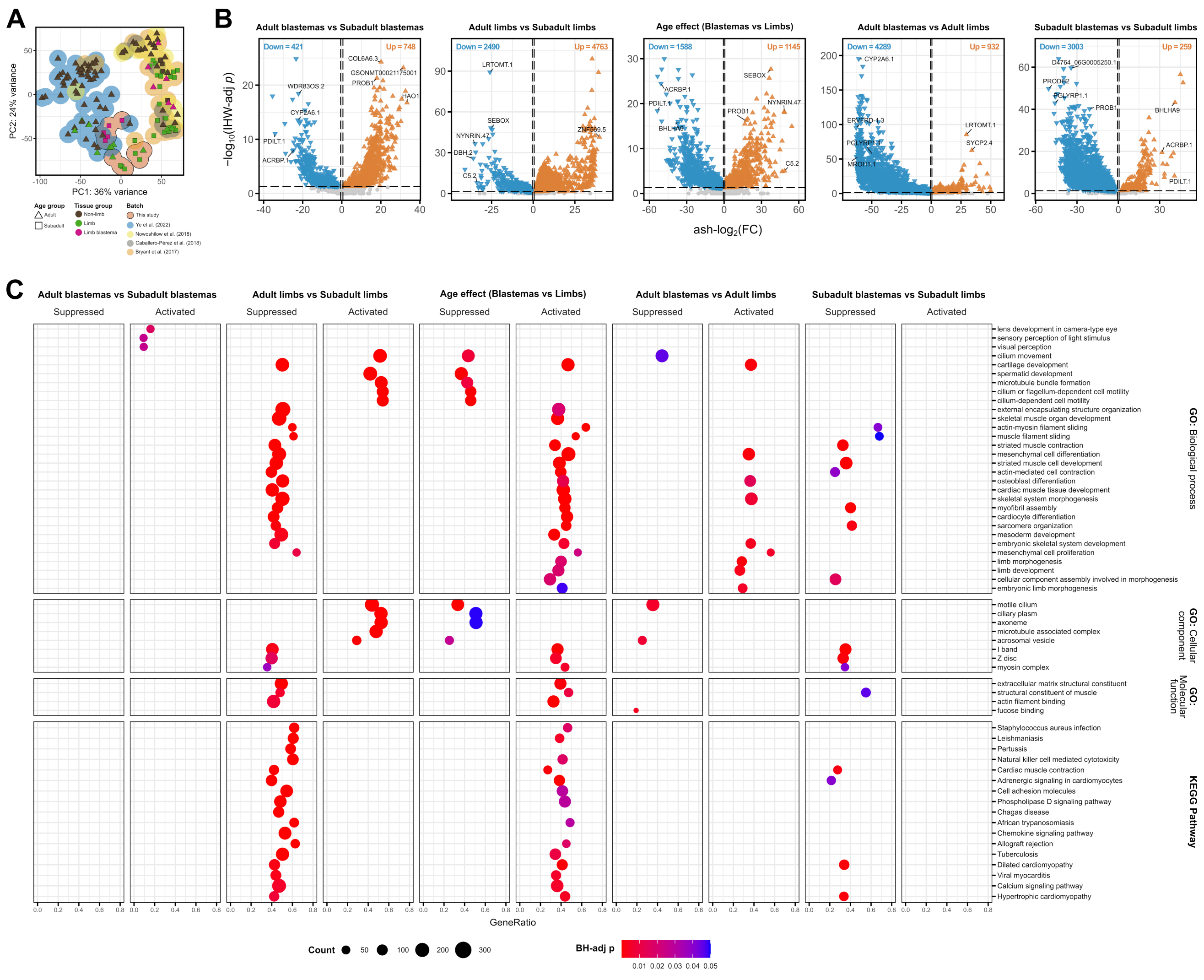

Figure S5. Gene expression differences between global axolotl samples of distinct age groups and between blastemas and limb tissues (Salmon quantification). (A) Principal component analysis (PCA) plot of gene counts (Salmon) after variance stabilizing transformation (VST). **(B)** Volcano plots based on Salmon quantification show the gene expression differences between adult and subadult blastemas, between adult and subadult limbs, the age effect differences between these tissues, between adult blastemas and adult limbs, and between subadult blastemas and subadult limbs. Genes with significantly upregulated (ash-$\log_2$(FC) > $\log_2$(1.5), IHW-adj p < 0.05) or downregulated (ash-$\log_2$(FC) < $-\log_2$(1.5), IHW-adj p < 0.05) expression (DEGs) are represented by orange and blue triangles, respectively. **(C)** Dotplot shows the significantly activated or suppressed gene ontology (GO) terms and Kyoto Encyclopedia of Genes and Genomes (KEGG) pathways identified for these contrasts contrast by fast gene set enrichment analysis (FGSEA). SA: subadult, A: adult, L: limb, B: limb blastema.

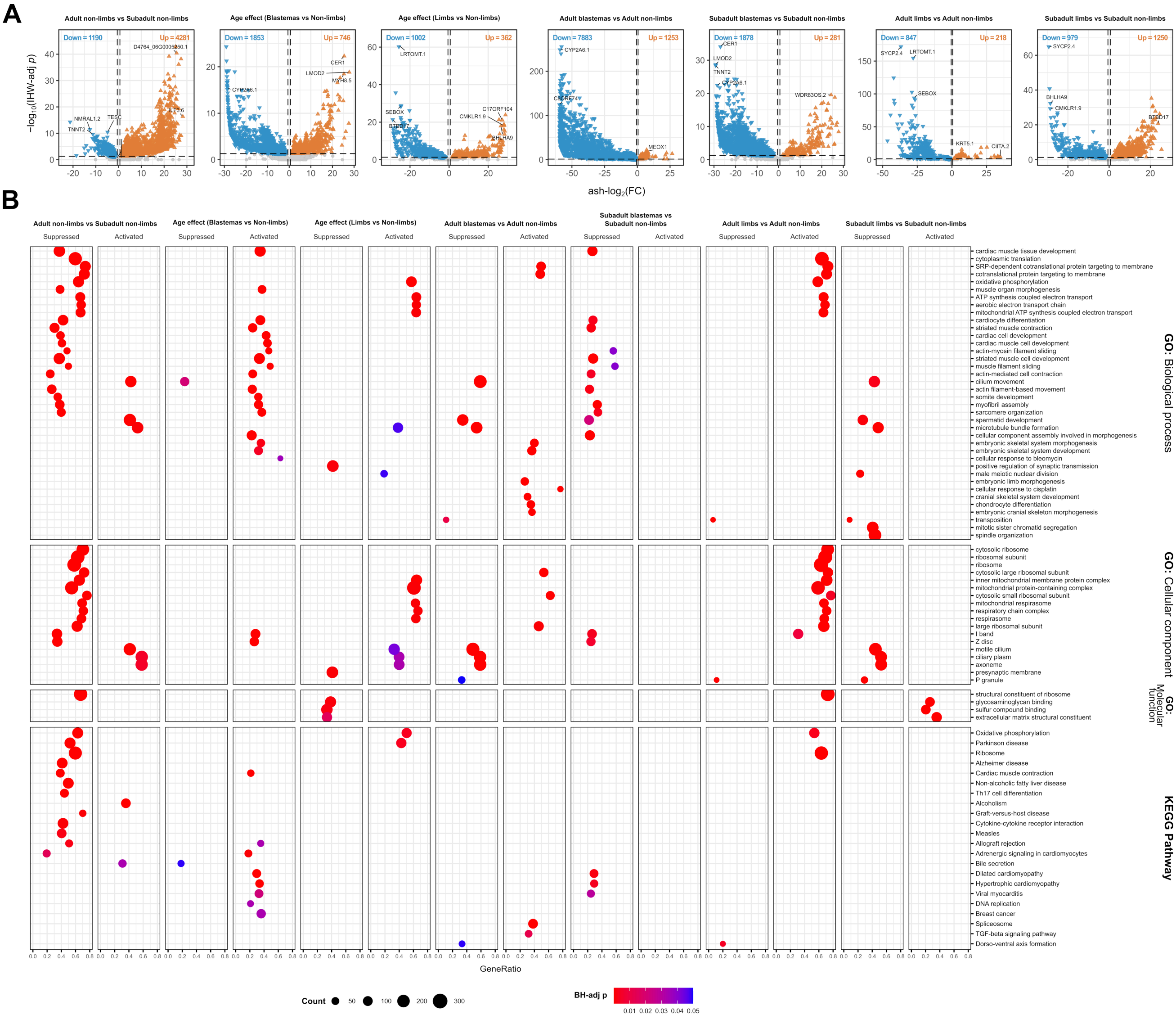

Figure S6. Gene expression differences between global axolotl samples of non-limb tissues and limbs and blastemas of distinct age groups (Salmon quantification). (A) Volcano plots based on Salmon quantification show the gene expression differences between adult and subadult non-limbs and the age effect differences between non-limbs and blastemas or limbs. Other tissue and age group contrasts involving non-limb tissues are also included. Genes with significantly upregulated (ash-$\log_2$(FC) > $\log_2$(1.5), IHW-adj p < 0.05) or downregulated (ash-$\log_2$(FC) < $-\log_2$(1.5), IHW-adj p < 0.05) expression (DEGs) are represented by orange and blue triangles, respectively. **(B)** Dotplot shows the significantly activated or suppressed gene ontology (GO) terms and Kyoto Encyclopedia of Genes and Genomes (KEGG) pathways identified for these contrasts by fast gene set enrichment analysis (FGSEA). SA: subadult, A: adult, L: limb, B: limb blastema, NL: non-limb.

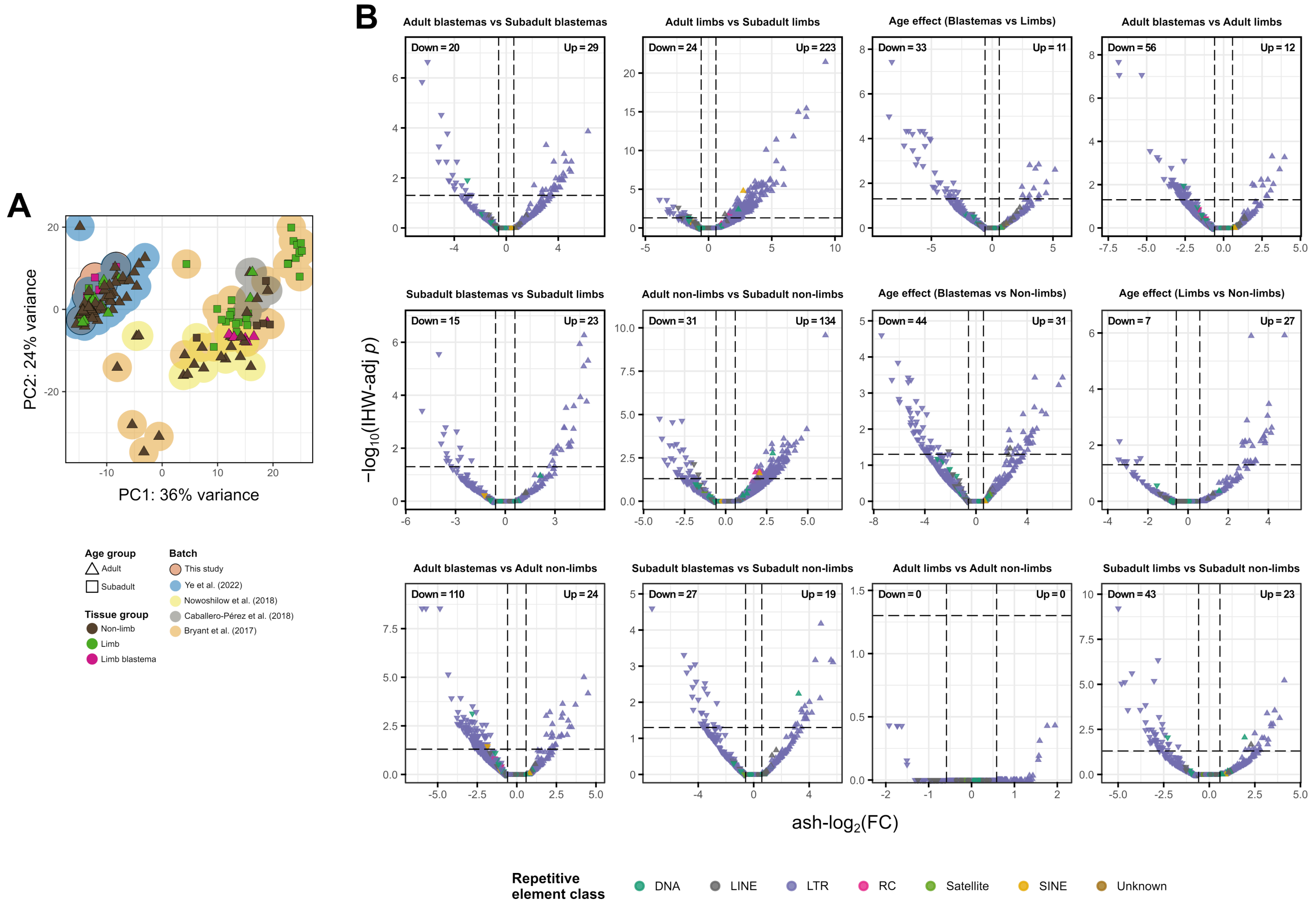

Figure S7. Effects of adulthood on the repetitive element expression changes in axolotl blastemas, limbs and non-limb tissues (ExplorATE quantification). (A) Principal component analysis (PCA) plot of repetitive element subfamily counts (ExplorATE) after variance stabilizing transformation (VST). **(A)** Volcano plots based on ExplorATE quantification show the repetitive element (RE) expression differences between age groups for blastemas and limbs, age effect differences between these tissues, as well as the contrasts between adult blastemas and adult limbs, and between subadult blastemas and subadult limbs. Other tissue and age group contrasts involving non-limb tissues are also included. REs with upregulated (ash-$\log_2$(FC) > $\log_2$(1.5)) or downregulated (ash-$\log_2$(FC) < $-\log_2$(1.5)) expression are represented by up-pointing and down-pointing triangles, respectively. Triangle color represents the RE class. SA: subadult, A: adult, L: limb, B: limb blastema, NL: non-limb.

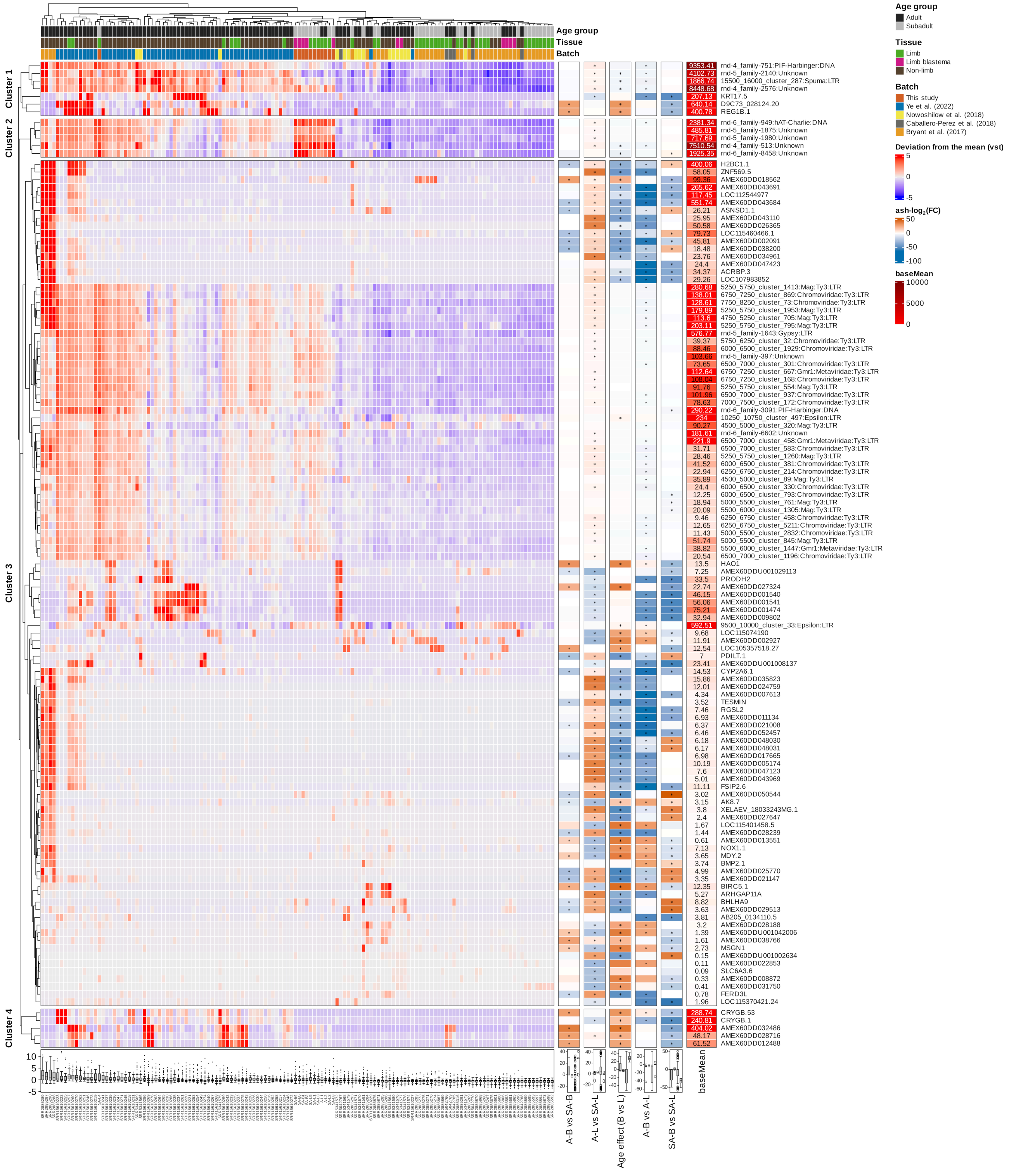

Figure S8. Clustering of genes and repetitive element subfamilies based on their expression in global samples of the axolotl's regenerating limb. (A) Heatmap of the deviation from the mean of each VST-normalized gene/RE count (TEcount), split into four clusters by the global dendrogram. Columns with color-coded ash-log2(FC) by contrast (∗: IHW-adj p < 0.05) are shown on the right for each gene/RE. These columns correspond to the contrasts between age groups for blastemas and limbs, age effect differences between these tissues, as well as the contrasts between adult blastemas and adult limbs, and between subadult blastemas and subadult limbs (Figures 3B-C and 4A). SA: subadult, A: adult, L: limb, B: limb blastema.

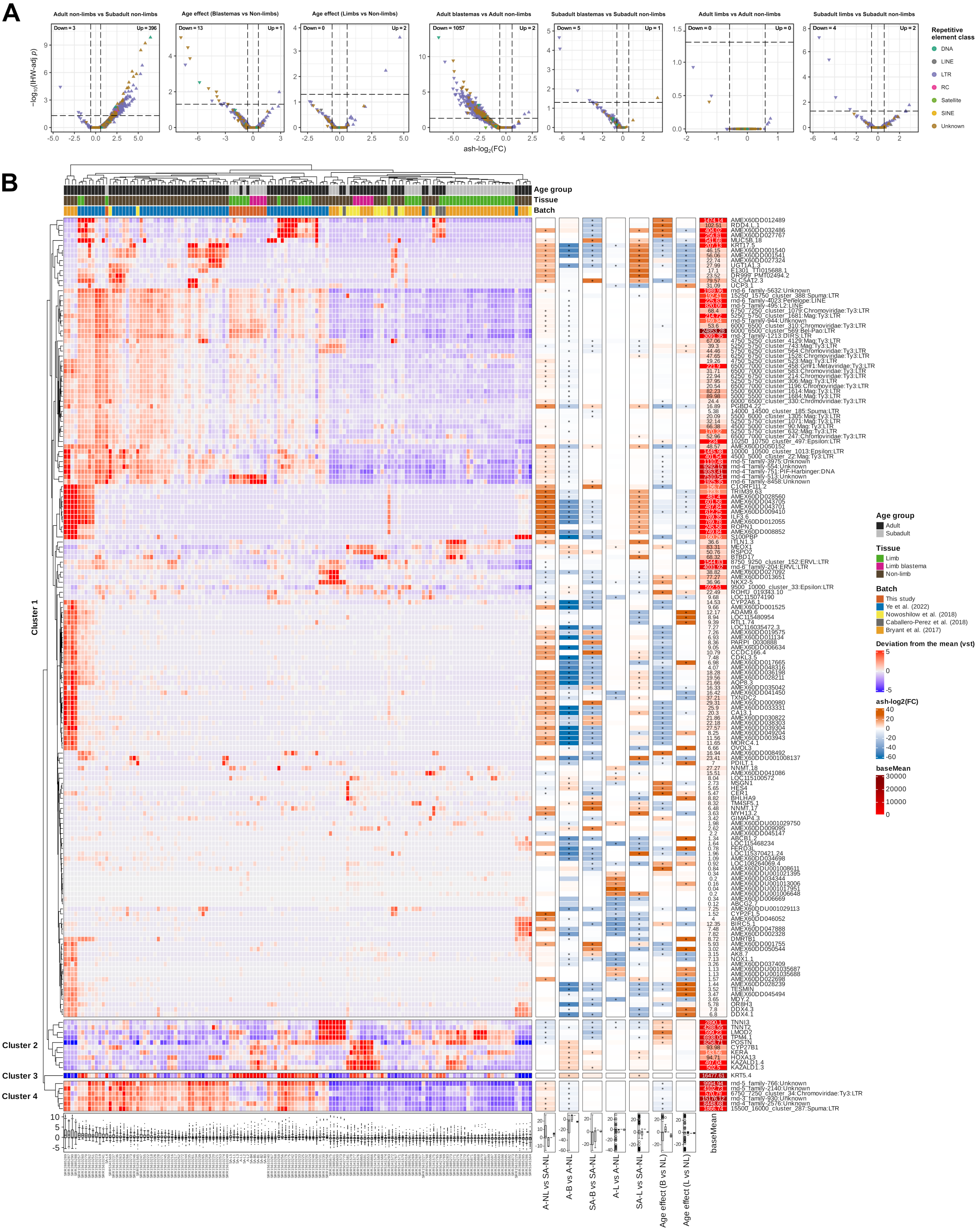

Figure S9. Clustering of genes and repetitive elements based on their expression in global samples of axolotl blastemas, limbs and non-limb tissues. (A) Volcano plots based on TEcount quantification show the repetitive element (RE) expression differences between adult and subadult non-limbs and the age effect differences between non-limbs and blastemas or limbs. Other tissue and age group contrasts involving non-limb tissues are also included. REs with upregulated (ash-$\log_2$(FC) > $\log_2$(1.5)) or downregulated (ash-$\log_2$(FC) < $-\log_2$(1.5)) expression are represented by up-pointing and down-pointing triangles, respectively. Triangle color

represents the RE class. **(B)** Heatmap of the deviation from the mean of each VST-normalized gene/RE count (TEcount), split into four clusters by the global dendrogram. Columns with color-coded ash-log2(FC) by contrast ($*$: IHW-adj $p < 0.05$) are shown on the right for each gene/RE. These columns correspond to the contrasts between tissue and age group contrasts involving non-limb tissues (S4A and S9A Figures). SA: subadult, A: adult, L: limb, B: limb blastema, NL: non-limb.

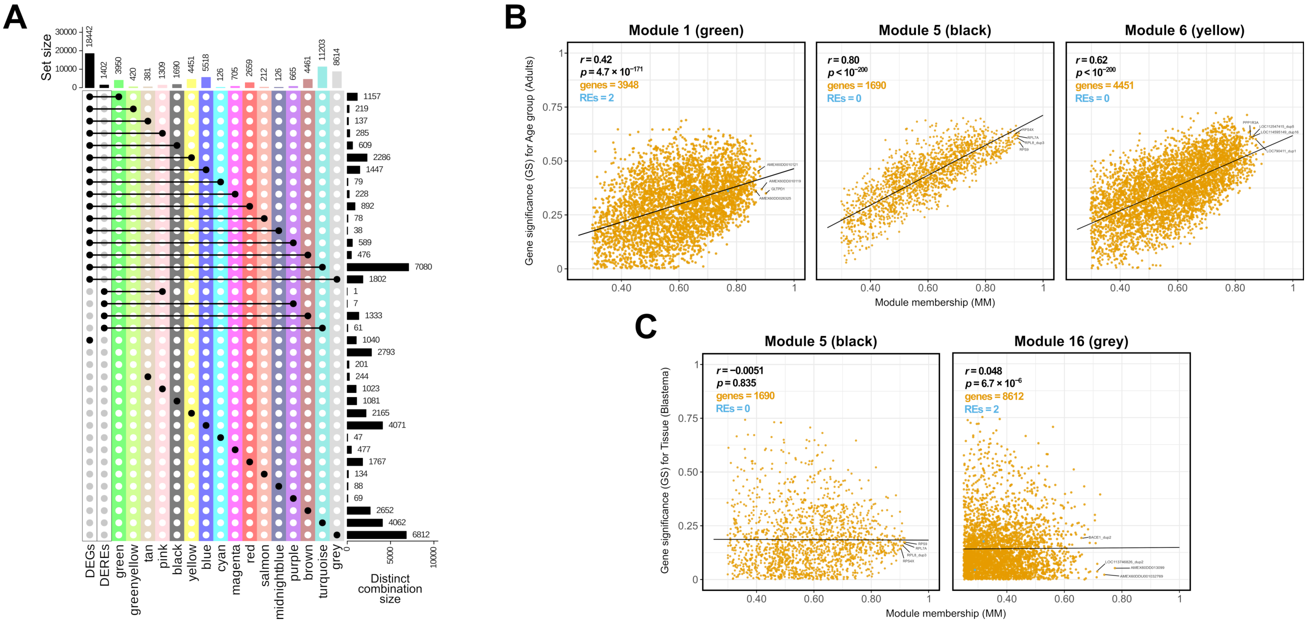

Figure S10. **Gene and repetitive element significance and membership in coexpression modules associated with adulthood and regeneration in the axolotl. (A)** UpSet plot of the intersections between module genes/REs and the complete set of differentially expressed genes and REs detected previously through differential expression analysis. **(B)** Scatterplots of gene significance (GS) for the age group trait versus module membership (MM) in the modules with highest correlation with age group. **(C)** Scatterplots of GS for the limb blastema tissue versus MM in the modules with highest correlation with this trait.

The following Supporting information is available from the Wiley Online Library or from the author:

Table S1. Metadata of the 136 RNA-seq samples of axolotl tissues analyzed in this study.

Table S2. Repetitive element genomic context annotation counts. The table lists as columns all the repetitive element subfamilies detected as differentially expressed (DEREs; |ash-log_2(FC)| > log_2(1.5), IHW-adj *p* < 0.05), classified by the quantification method and the differential expression contrast. The numbers correspond to the count of DEREs that overlapped with each genomic/genic feature (rows) per contrast, direction of differential expression, and clustering.

Table S3. Repetitive element genomic context annotation totals. The table lists the total count and percentage of differentially expressed repetitive element subfamilies (DEREs) per type of genomic/genic feature overlap, contrast, direction of differential expression, and clustering.

Table S4. List of the top differentially expressed genes and repetitive elements. The genes and or repetitive element subfamilies with the 15 highest and 15 lowest ash-log_2(FC) values for each of the contrasts of interest are included, as well as their WGCNA module.

Table S5. Metadata of primers used in qPCR validation.
